# Recapitulation of developmental mechanisms to revascularize the ischemic heart

**DOI:** 10.1172/jci.insight.96800

**Published:** 2017-11-16

**Authors:** Karina N. Dubé, Tonia M. Thomas, Sonali Munshaw, Mala Rohling, Paul R. Riley, Nicola Smart

**Affiliations:** 1 UCL Institute of Child Health, London, United Kingdom.; 2 British Heart Foundation Centre of Regenerative Medicine, Department of Physiology, Anatomy and Genetics, University of Oxford, Oxford, United Kingdom.

**Keywords:** Cardiology, Adult stem cells, Heart failure, endothelial cells

## Abstract

Restoring blood flow after myocardial infarction (MI) is essential for survival of existing and newly regenerated tissue. Endogenous vascular repair processes are deployed following injury but are poorly understood. We sought to determine whether developmental mechanisms of coronary vessel formation are intrinsically reactivated in the adult mouse after MI. Using pulse-chase genetic lineage tracing, we establish that de novo vessel formation constitutes a substantial component of the neovascular response, with apparent cellular contributions from the endocardium and coronary sinus. The adult heart reverts to its former hypertrabeculated state and repeats the process of compaction, which may facilitate endocardium-derived neovascularization. The capacity for angiogenic sprouting of the coronary sinus vein, the adult derivative of the sinus venosus, may also reflect its embryonic origin. The quiescent epicardium is reactivated and, while direct cellular contribution to new vessels is minimal, it supports the directional expansion of the neovessel network toward the infarcted myocardium. Thymosin β4, a peptide with roles in vascular development, was required for endocardial compaction, epicardial vessel expansion, and smooth muscle cell recruitment. Insight into pathways that regulate endogenous vascular repair, drawing on comparisons with development, may reveal novel targets for therapeutically enhancing neovascularization.

## Abstract 2

Embryonic mechanisms are redeployed to revascularize the ischemic heart, with contributions primarily from the endocardium and coronary sinus and processes that require thymosin β4.

## Introduction

Timely reperfusion therapy limits the duration of ischemia and consequently the extent of myocardial loss, adverse remodeling, and progression to heart failure. However, the “no-reflow phenomenon,” resulting from compromised microvascular integrity and patency, leads to incomplete reperfusion, despite restoring flow to the epicardial vessels (1). Angiogenic growth factor therapy has demonstrated minimal efficacy in clinical trials (2, 3), and progenitor cell transplantation approaches are hindered by limited engraftment and trans-differentiation, although they have proven paracrine benefits (4). These failures betray both a lack of understanding of the endogenous neovascularization that occurs in the heart in response to ischemic injury and an appreciation of the processes that underlie the establishment of a hierarchical vascular network, as occurs during development. Most studies to date quantify capillary density without distinguishing de novo from preexisting vessels and give little consideration to the cellular sources and mechanisms that lead to remodeling and expansion of the coronary network. Thus far, it has been assumed that new vessels derive exclusively via angiogenesis (5), alongside collateral growth (6).

Coronary vessel development occurs via vasculogenesis, angiogenesis, and arteriogenesis, with compartmentalized contribution of coronary endothelial cells (ECs) from the sinus venosus (7) and endocardium (8) to the dorsal/lateral and ventral/midline aspects, respectively (9). The sinus venosus is a transient structure that serves to return venous blood to the developing heart. At later embryonic stages, the right horn of the sinus venosus incorporates into the right atrium and superior vena cava, whereas the left horn gives rise to the adult coronary sinus and the oblique vein of Marshall (10). Beyond embryonic stages, the endocardium also contributes ECs to enable perinatal expansion of the capillary network. A distinct mechanism was proposed for this, although angiogenesis cannot be excluded; during compaction, endocardial cells on the trabeculated surfaces become trapped within the muscle and coalesce to rapidly form new vessels and allow perfusion of the surrounding myocardium (11). A further, modest contribution (5%–15% of ECs) derives from a subcompartment of the proepicardium, and these are distributed throughout the ventricular walls (9, 12). Coronary smooth muscle cells (coVSMCs) and adventitial fibroblasts from the proepicardium migrate in to support the forming vessels of the ventricular walls (13).

Although a previous study proposed a mesenchymal-to-endothelial transition from fibroblasts as the contributing source of de novo ECs after myocardial infarction (MI) (14), it has recently been argued that all new coronary vessels in the injured heart derive from preexisting ECs (15). However, the exact sources of EC contribution cannot be unambiguously delineated in the adult, and the field is currently limited to descriptive studies; the lineage-specific markers employed effectively in embryonic studies are either silenced in the adult heart or expressed in a pan-endothelial manner, regardless of anatomical location or embryonic origin. Using *Pdgfb*CreERT2 (16) genetic lineage tracing, with a pulse-chase strategy, to label existing ECs before MI, we report here that a substantial proportion of vessels arise de novo, potentially from reactivated developmental sources. An apparent contributory source was the endocardium, consistent with an observed hypertrabeculation upon injury and subsequent compaction, leading to a progressive increase in the incidence of subendocardial vessels. In addition, we observed overt angiogenic sprouting from the coronary sinus vein, which was not observed in any other vessels of the heart, possibly reflecting an imprinted memory of its embryonic role as the sinus venosus. The process of endocardial neovessel formation was dependent upon the actin-monomer-binding protein thymosin β4 (Tβ4), previously implicated in coronary (17) and systemic (18) vessel development. Thus, our observations suggest a recapitulation of primary sources of embryonic and early postnatal coronary vessel development as endogenous repair mechanisms, which might restore a functional coronary vasculature after injury.

## Results

### Acute neovascularization after MI occurs via angiogenic and nonangiogenic mechanisms.

**Figure 1:**
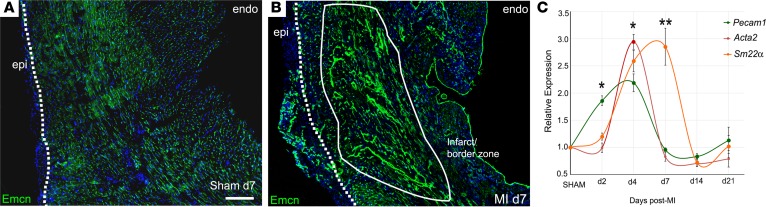
The endogenous neovascular response after myocardial infarction. Immunostained transverse heart sections reveal considerable expansion of the capillary network by day 7 after infarction (**B**; compared with sham, **A**). Note the robust upregulation of Emcn, particularly in the endocardium, and the de novo capillary network that forms within the expanded epicardium (dotted line indicates epicardial-myocardial boundary; solid line indicates infarcted myocardium; representative of *n* = 15 day 7 MI hearts and *n* = 4 day 7 sham hearts). Scale bar: 200 μm (**A** and **B**). By qRT-PCR, upregulation of endothelial genes, exemplified by *Pecam1*, precedes induction of smooth muscle genes, *Acta2* and *Sm22α* (**C**) (mean ± SEM; *n* = 4 hearts per time point). Two-tailed Kruskal-Wallis nonparametric test with Dunn’s post-hoc test for multiple comparisons; *P ≤ 0.05, **P ≤ 0.01.

We initially examined the vascular response in murine hearts following permanent ligation of the left anterior descending coronary artery (LAD) across a time course. In transverse sections, by day 7, a striking expansion of the endomucin-labeled (Emcn-labeled) capillary network into the infarct and border zone was evident, compared with sham controls, with extensive branching and many long vessels extending from the border zone into the infarcted myocardium (Figure 1, A and B). An additional capillary network, discrete from the infarct region, developed de novo within the expanded epicardium, and, while a modest degree of epicardial expansion resulted from sham injury (Figure 1A), a robust expansion and coincident new vessel formation were features of the ischemic heart after MI (Figure 1B). Emcn levels were markedly elevated both in new and preexisting vessels and were strongly upregulated in cells of the endocardium (Figure 1B). We assessed expression of additional vascular markers by quantitative RT-PCR (Figure 1C); the pan-EC marker, *Pecam1*, increased 2-fold as early as day 2, followed by significant increases in coVSMC markers, *Acta2* and *Sm22a*, which peaked at day 4 and day 7, respectively, before returning to baseline by day 14. To further understand the processes of neovascularization, morphological alterations and expression of EC (Emcn, PECAM1) and coVSMCs markers (smooth muscle myosin heavy chain [SM-MHC]) were examined by immunofluorescence over a time course after MI (Supplemental Figure 1; supplemental material available online with this article; https://doi.org/10.1172/jci.insight.96800DS1). The early elevation in PECAM1 (and Emcn) by day 2 reflected upregulated expression per cell, with no indication of additional ECs at this stage. A further increase in signal intensity by day 4 accompanied visible sprouting. Thereafter, reduced expression reflected both a notable decline in expression per cell, between day 4 and day 7 in the sprouting vessels, as well as an appreciable loss overall in the number of capillaries within the ischemic tissue (compare time series in Supplemental Figure 1, A–E and K–O). After expansion into the infarct/border zone, the capillaries progressively enlarged and arterialized by day 7. Very few capillary ECs in the border zone were found to be proliferating at day 7 (1.2% ± 0.3%; based on 3 border zone fields; *n* = 6). The preexisting arteries and arterioles of the healthy myocardium adjacent to the infarct were entirely nonproliferative (Supplemental Figure 2A), whereas coVSMCs of the “de novo” arterioles within the expanded epicardium showed high levels of proliferation (29.5% ± 1.7% Ki67^+^ coVSMCs, Supplemental Figure 2A; based on 3 epicardial fields per heart; *n* = 6 hearts). Rare, proliferating ECs were notably detected in the medium-large veins below the activated epicardium (Supplemental Figure 2B), and veins, but not arteries, within the border zone, expressed endoglin/CD105 (Supplemental Figure 2, C–E), previously implicated in extracellular matrix (ECM) remodeling, intimal angiogenesis, and tumor neovascularization (19), suggesting a higher propensity for venous, compared with arterial, sprouting.

**Figure 2:**
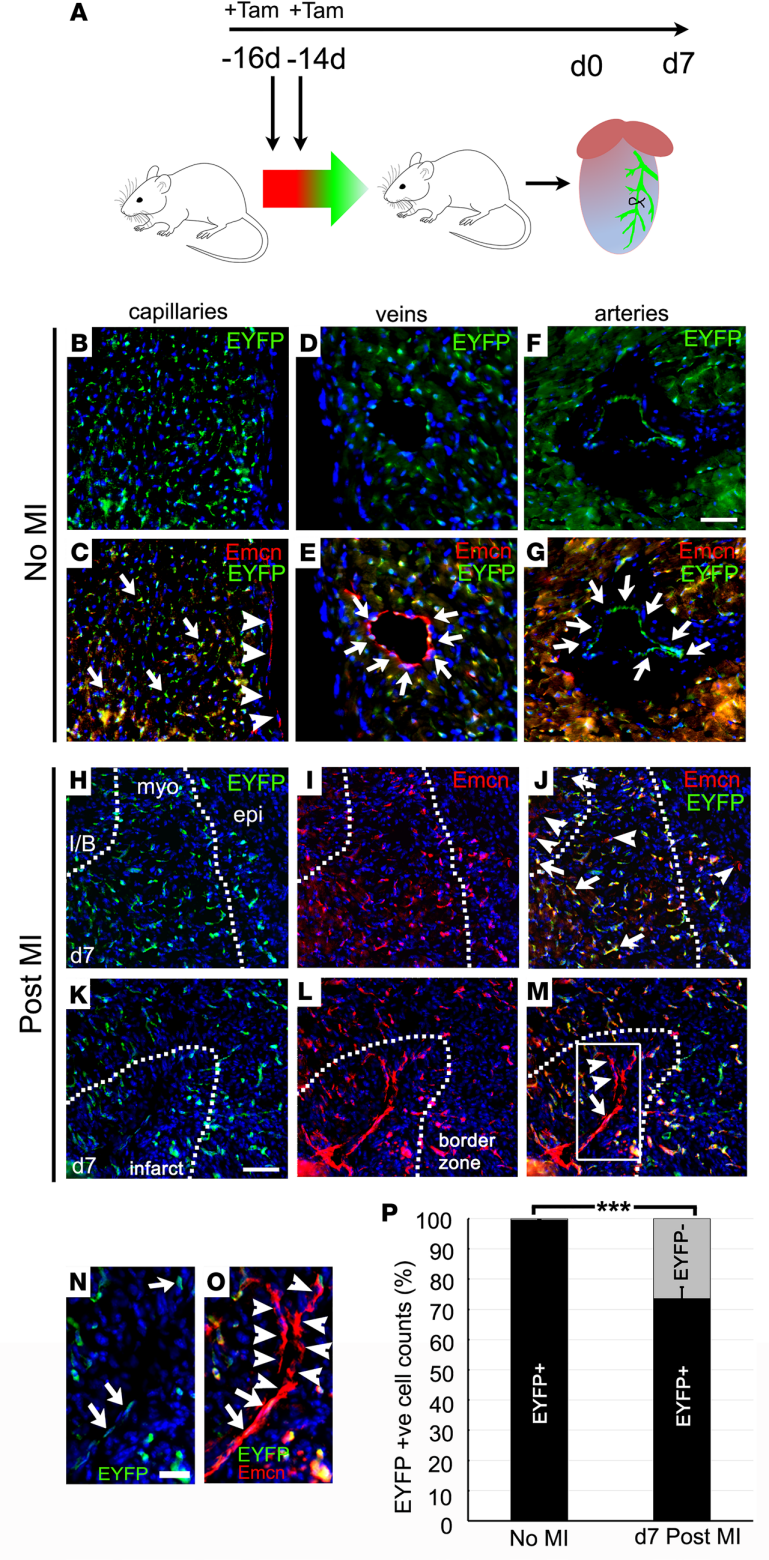
Lineage-tracing neovascularization in the infarcted heart. *Pdgfb*CreERT2; R26R-EYFP mice were injected with 2 doses of 2 mg tamoxifen to prelabel coronary endothelial cells, 2 weeks prior to myocardial infarction (**A**), and assessed before/after infarction by immunostaining. In noninfarcted mice, capillary (**B** and **C**), venous (**D** and **E**), and arterial (**F** and **G**) endothelial cells were efficiently labeled with EYFP (EYFP^+^ cells indicated with arrows); only the endocardium remained 100% unlabeled with the reporter (arrowheads in **C**). In the border zone after infarction (day 7), 73.6% ± 3.4% of capillary endothelial cells were EYFP^+^ (**H**–**M**; box in **M** enlarged in **N** and **O**; arrows indicate EYFP^+^ cells), thus preexisting or derived via angiogenesis from existing endothelium. However, 26.4% ± 3.4% of infarct/border zone endothelial cells were EYFP^–^ (**H**–**J** and **P**), including long capillaries extending into the infarct (**K**–**O**; arrowheads indicate EYFP^–^ ECs). EYFP^–^ cells were *Pdgfb*^–^ prior to injury and thus newly derived, either via vasculogenesis from a nonendothelial source or from the endocardium. MI, myocardial infarction; I/B, infarct/border zone; myo, myocardium; epi, epicardium; Tam, tamoxifen. Scale bars: 20 μm (**B**–**G**); 50 μm (**H**–**M**); 20 μm (**N** and **O**). *n* = 4 injured hearts and *n* = 3 uninjured hearts; quantification from 4 capillary fields, 6 arteries, and 6 veins per uninjured heart and from 4 border zone regions each from 3 sections, approximately 100 μm apart, per inured heart. Mean ± SEM; 2-tailed Student’s *t* test; ***P ≤ 0.001.

Seeking further insight into mechanisms and sources of new vessels, we used a *Pdgfb*CreERT2; R26R-EYFP reporter cross (16, 20) to prelabel all coronary ECs by tamoxifen injection of adult mice, 2 weeks prior to MI (Figure 2A). This regimen ensured that tamoxifen, with a half-life of approximately 12 hours in mice (21), was depleted from the circulation prior to formation of any new, injury-induced ECs, preventing their labeling. In noninfarcted mice, ECs were very efficiently labeled (98.9% ± 0.2%, 99.5% ± 0.4%, and 99.2% ± 0.1% of capillary, venous, and arterial ECs, respectively, were EYFP^+^; Figure 2, B–G). Only ECs of the endocardium were unlabeled by this approach (Figure 2C). Following MI, 73.6% ± 3.4% of capillary ECs within the border zone were EYFP^+^, identifying this population as preexisting or derived via angiogenic sprouting of preexisting vessels. However, 26.4% ± 3.4% of infarct/border ECs were EYFP^–^ (Figure 2, H–J and P), notably including the longitudinal capillaries that extended into the infarct region (Figure 2, K–O). Interestingly, some vessels were found to comprise both EYFP^+^ and EYFP^–^ ECs (Figure 2, N and O), suggesting that unlabeled progenitors can interact with, and incorporate into, sprouting angiogenic vessels. In remote regions, 99.4% ± 0.3% of capillary ECs remained EYFP^+^ after MI (data not shown). We concluded that EYFP^–^ cells, contributing to border zone vessels, did not express the *Pdgfb*-dependent endothelial reporter prior to MI and may be derived either from endocardial cells, the only unlabeled EC source within the heart, or via vasculogenesis from a non-EC origin.

### Endocardial remodeling contributes new vessels after MI.

**Figure 3:**
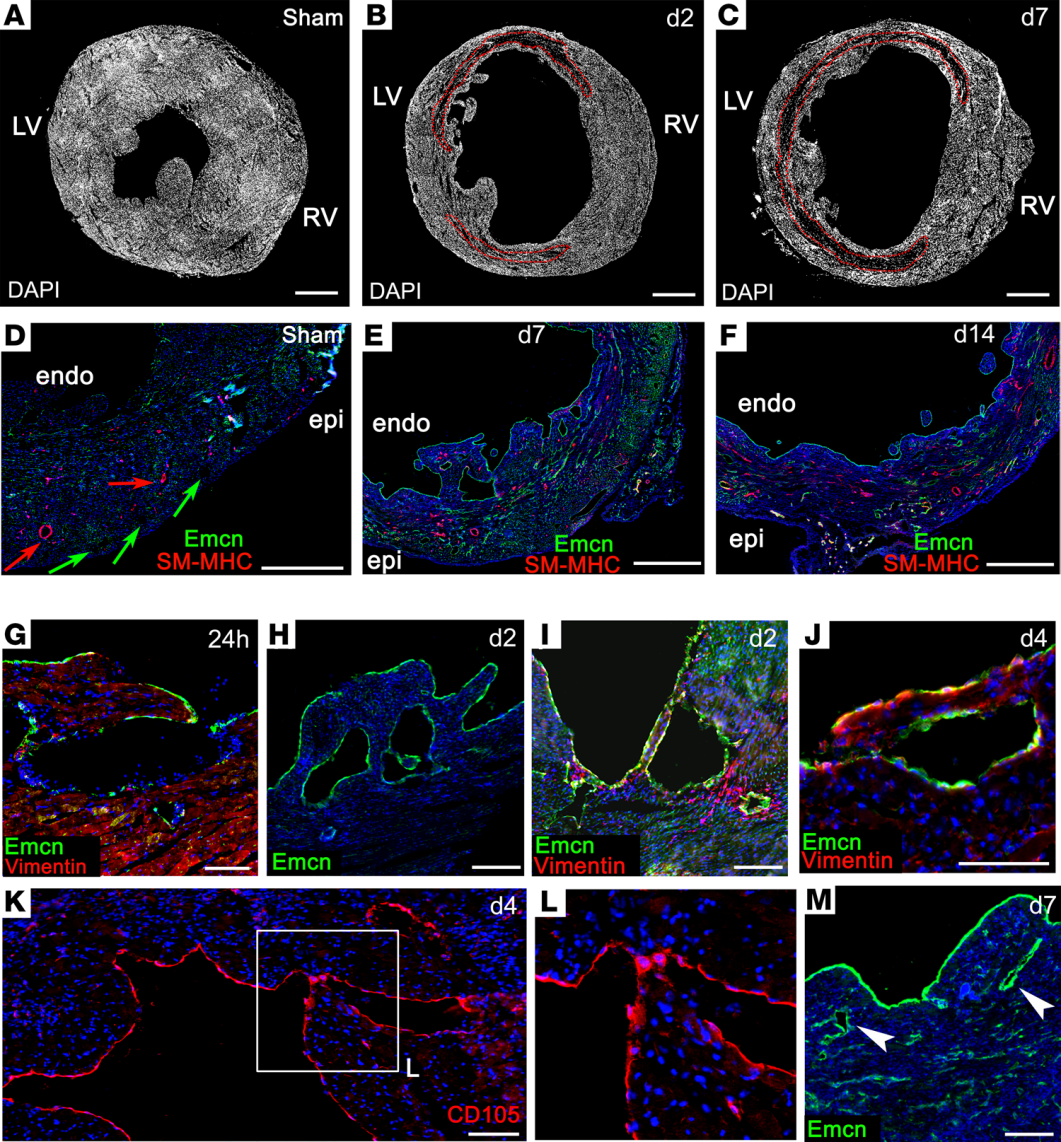
Remodeling of the endocardium after myocardial infarction. Immunostaining revealed increased trabeculation of the endocardial surface following MI: a representative sham-operated heart (**A**), compared with MI hearts after 2 (**B**) and 7 (**C**) days. Whereas, in the uninfarcted heart, large coronary arteries (red arrows) and veins (green arrows) were restricted to the epicardial side of the ventricle (**D**), new vessels appeared on the endocardial side of the infarct (**E** and **F**), coincident with compaction of trabeculae. Altered endocardial morphology was detected as early as 24 hours, with formation of cavities and finger-like protrusions (**G**–**I**). Coalescence of endocardial cells and trabecular compaction between days 4 and 7 (**J**–**M**; box in **K** enlarged in **L**) coincided with appearance of subendocardial vessels (arrowheads, **M**). LV, left ventricle; RV, right ventricle; epi, epicardium; endo, endocardium. Scale bars: 1 mm (**A**–**F**); 100 μm (**G**, **K**, and **J**); 200 μm (**H**, **I**, and **M**). The boxed area in **K** is magnified 2-fold in **L**. Representative of 24 hours: *n* = 5 (2 sham); 2 days: *n* = 6 (3 sham); 4 days: *n* = 5 (2 sham); 7 days: *n* = 15 (4 sham); 14 days: *n* = 5 (2 sham).

**Figure 4:**
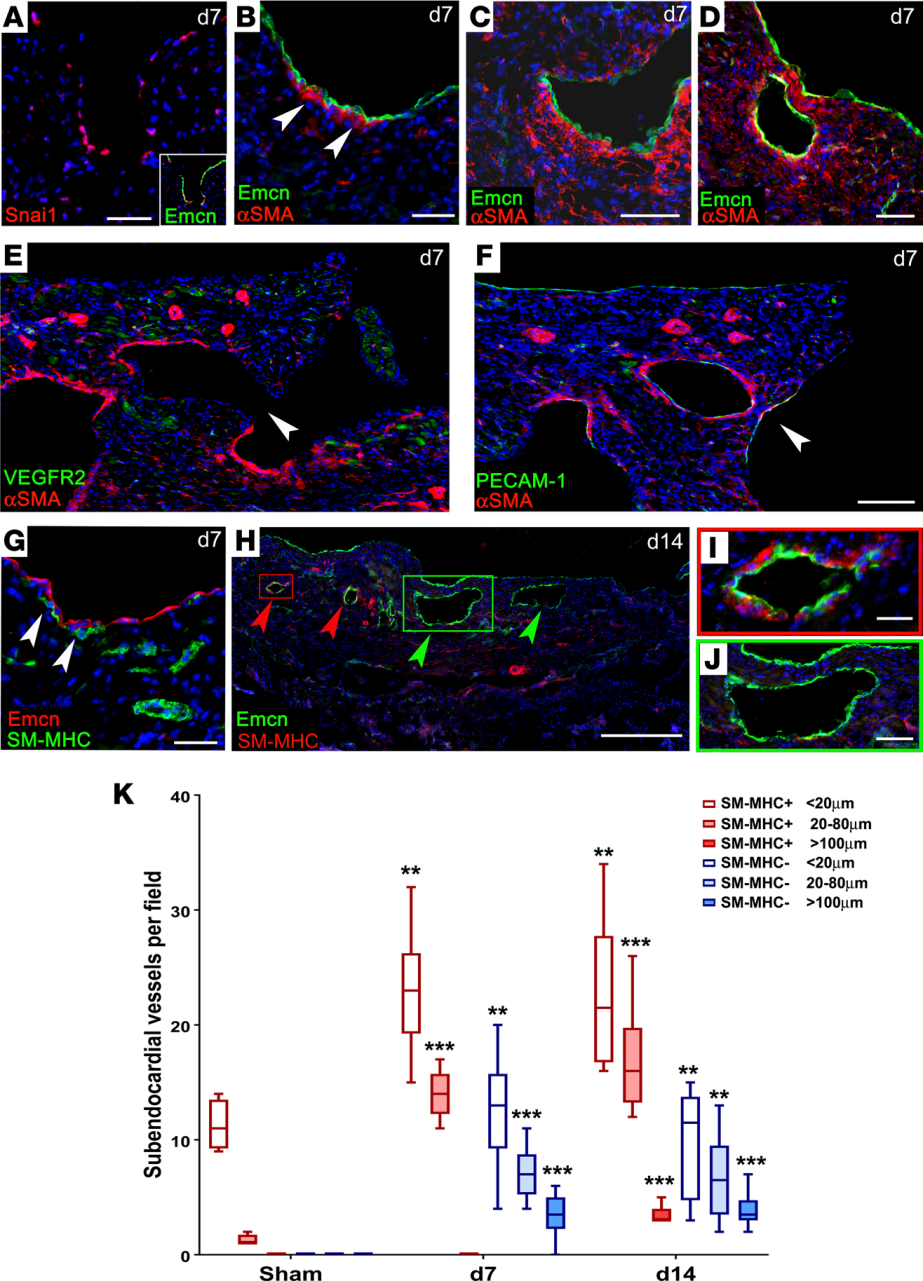
Endocardial remodeling contributes new subendocardial vessels. Detection of αSMA^+^ cells by immunostaining suggested either recruitment of smooth muscle precursors or endothelial-to-mesenchymal transition (**A**–**D**, arrowheads in **B**), consistent with expression of Snai1, in endocardial cells (**A**). Serial sections, approximately 50 μm apart, revealed progressive closure of subendocardial vessels: in (**E**), the lumen on the endocardial surface was incompletely enclosed (arrowhead) and only the enclosed side had αSMA support, whereas, in a more apical section (**F**), the equivalent lumen was fully enclosed (arrowhead) and surrounded by αSMA^+^ mural cells. Later detection of SM-MHC^+^ cells, from 7–14 days (arrowheads, **G**), confirmed a mature smooth muscle phenotype (**G**). By day 14, some subendocardial vessels were fully enclosed by SM-MHC^+^ cells (red arrowheads, **H**; enlarged in **I**), whereas larger vessels remained devoid of VSMC support (green arrowheads, **H**; enlarged in **J**). Scale bars: 100 μm (**A**–**D**); 500 μm (**E**, **F**, and **H**); 50 μm (**G** and **J**); 10 μm (**I**). An increase in small/medium SM-MHC^+^ and SM-MHC^–^ vessels was observed by day 7 and an increase in large SM-MHC^+^ vessels was observed by day 14. Representative of day 7: *n* = 15 (4 sham); day 14: *n* = 5 (2 sham). (**K**) Vessel count number per 3-mm segment, between endocardium and infarct; sham: *n* = 4; day 7: *n* = 8; day 14: *n* = 8. Box-and-whisker plots show mean ± minimum/maximum. 1-way ANOVA with Bonferroni correction; **P ≤ 0.01, ***P ≤ 0.001 versus sham.

We therefore considered the endocardium as a neovessel source after MI, given its contributions to the forming coronary vessels via developmental angiogenesis and trabeculation/compaction. We observed no overt endocardial cell sprouting and the rates of proliferation were relatively low in the endocardium, compared with either the infarct region or the epicardium (Supplemental Figure 3C). Rather, the most striking observation was an induced remodeling in the form of hypertrabeculation of the endocardial surface. At the level of whole-heart sections, trabeculae appeared on the endocardium of the lateral left ventricular (LV) wall within 2 days after MI (Figure 3, A and B) and further remodeling led to extensive trabeculation, typically peaking at day 7 (Figure 3C). By contrast, sham control hearts did not reveal any distinctive endocardial protrusions (fixed in diastole for maximal dilatation; Figure 3, A and D). The Emcn-lined lumina, which appeared in the subendocardium by day 7 after MI, were also striking (Figure 3E; note also, the strong upregulation of Emcn in the endocardium, relative to sham), and, between day 7 and day 14, an increase in medium-sized vessels appeared below the endocardial surface, coincident with compaction of the trabeculated surface (Figure 3F). In stark contrast, no medium or large vessels, and very few arterioles/venules, were ever detected on the subendocardial side of the uninjured heart (Figure 3D and Figure 4K). The earliest indication of altered endocardial morphology was detected at 24 hours, with the formation of cavities and finger-like protrusions (Figure 3G). The morphogenetic processes underlying formation of trabeculae are currently unclear. Small clusters of apoptotic cardiomyocytes (CC3^+^) were observed, adjacent to forming protrusions (Supplemental Figure 3, A and B), although these were scarce (0.56% ± 0.07% at 24 hours; 0.23% ± 0.09% at 48 hours and declined further thereafter). Proliferation levels were lower in the subendocardium than in other regions of the heart (Supplemental Figure 3C). Thus, while localized proliferation and cell death cannot be excluded, the mechanism of trabeculation appears largely to proceed via morphological reorganization of the endocardial surface. From day 2, more definitive lumina, lined with Emcn^+^ cells, were found below the endocardium (Figure 3, H and I). Closer examination of cellular morphology revealed coalescing endoglin/CD105^+^ endocardial cells of the trabeculae (Figure 3, J–L) from day 4, and enclosed Emcn-lined structures, resembling vessels, were found <200 μm below the endocardial surface by day 7 (Figure 3M). The appearance of cells expressing mesenchymal markers, vimentin (Figure 3J) and αSMA, from day 7, emerging around the base of the forming trabeculae and lumina, is consistent with a possible endothelial-to-mesenchymal transition (EndMT) from the endocardium. This mechanism has been shown to contribute pericytes and coVSMCs to the developing heart (22), and, supporting the possibility of its recapitulation in adult, we observed expression of Snai1, a transcriptional repressor known to induce epithelial-to-mesenchymal transition (EMT) and EndMT, in endocardial cells at the base of forming trabeculae (Figure 4A), although definitive proof of EndMT is currently lacking. αSMA^+^ cells first appeared at the base of forming trabeculae (Figure 4B) and increased in number coincident with trabecular extension (Figure 4C), until they fully surrounded enclosed lumina (Figure 4D). By examining the equivalent region of LV endocardium in serial sections, taken approximately 50 μm apart, from the suture to the apex, it was possible to appreciate a progressive enclosure of subendocardial vessels after MI, an example of which is shown in Figure 4, E and F. In an apical-basal direction, we observed a trabecular lumen, with an aperture on the endocardial surface, in which only the enclosed side of the lumen had acquired mural cell support (Figure 4E); more basally, sections revealed an enclosed lumen, which was entirely surrounded by αSMA^+^ mural cells (Figure 4F). While αSMA does not distinguish mesenchymal cells from coVSMCs, the later detection of SM-MHC, a mature coVSMC marker, on cells associating with the endocardium on the ventricular side, implied either a transition toward, or recruitment of, bona fide coVSMCs (Figure 4G). By day 14, some subendocardial vessels were fully enclosed by SM-MHC^+^ cells (Figure 4, H and I). As shown in Figure 3D, Emcn and SM-MHC were expressed in a mutually exclusive pattern in the sham control heart, in veins and arteries, respectively. Thus, the concurrence of Emcn and SM-MHC supports their recent derivation and putative arterialization of vessels from the endocardium following injury (Figure 4I). Also of note, in many hearts at day 7, and as late as day 14, the largest subendocardial vessels remained devoid of coVSMC support (Figure 4, H and J); possibly reflecting a temporal delay in arterialization or a commitment to venous fate. The increased incidence of small/medium SM-MHC^+^ and Emcn^+^ (SM-MHC^–^) vessels underlying the endocardium was highly significant by day 7 and increased further by day 14 to include a small number SM-MHC^+^ vessels of >100 μm (quantified in Figure 4K).

### Molecular characterization of subendocardial vessels after MI.

**Figure 5:**
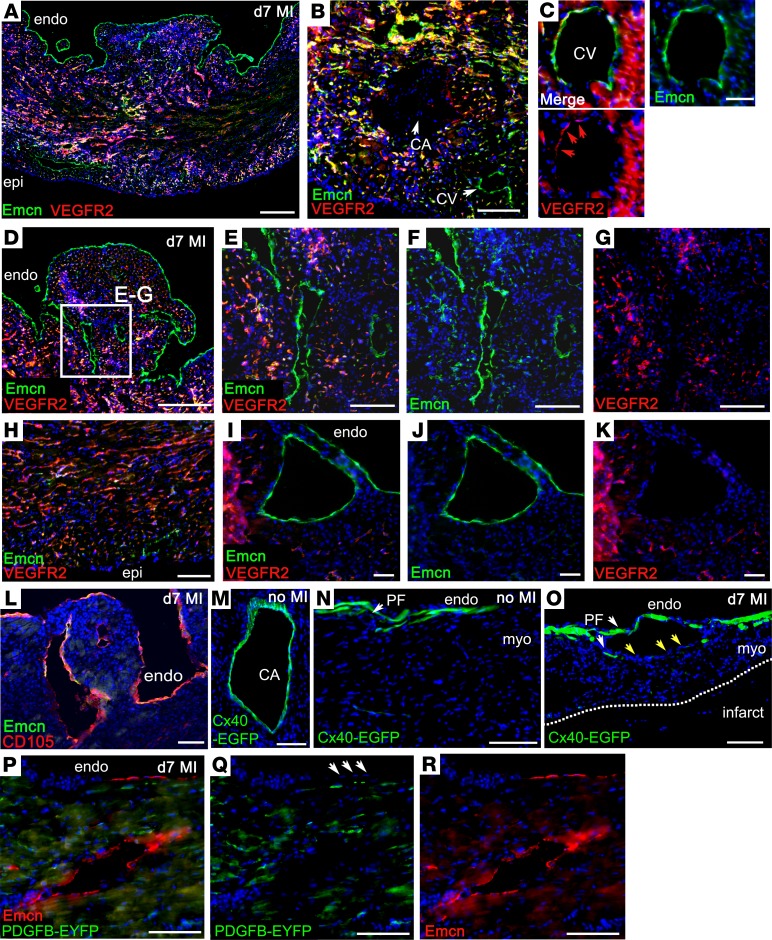
Marker profiling supports an endocardial origin of de novo subendocardial vessels. By immunostaining, VEGFR2 was expressed in capillaries (**A**) and veins weakly (**B** and **C**) but not arteries (arrowhead, **B**) or endocardium (**A** and **D**). Subendocardial vessels, formed after myocardial infarction (MI), did not express VEGFR2 (**D**, enlarged in **E**–**G**; **I**–**K**). Medium-power view of VEGFR2^+^ capillaries (**H**). Emcn (**A** and **D**–**J**) and endoglin/CD105 (**L**) were highly upregulated in endocardium and expressed in subendocardial vessels. Cx40-EGFP was expressed in preexisting coronary arteries (shown in uninjured heart, **M**), but not in de novo subendocardial vessels (**N**), consistent with a nonarterial origin. Cx40-EGFP is expressed in Purkinje fibers (white arrowheads, **N** and **O**), and these were displaced around forming subendocardial vessels in MI (yellow arrowheads, **O**) but not uninjured, hearts (**N**). In the *Pdgfb*CreERT2; R26R-EYFP reporter line, the endocardium (arrowheads, **Q**) and all endothelial cells of subendocardial vessels formed after MI were entirely negative for EYFP (**P**–**R**), confirming their derivation from a cell type that did not express *Pdgfb*/EYFP prior to MI. Representative of *n* = 15 WT MI hearts (*n* = 4 sham); *Pdgfb*CreERT2 × R26R-EYFP hearts: *n* = 4 MI hearts (*n* = 3 sham); Cx40-EGFP: *n* = 6 MI hearts (*n* = 3 sham). epi, epicardium; endo, endocardium; CA, coronary artery; CV, coronary vein; myo, myocardium; PF, Purkinje fiber. Scale bars: 200 μm (**A** and **D**); 100 μm (**L**, **M**, and **O**); 50 μm (**B**, **E**–**H**, **N**, and **P**–**R**); 20 μm (**C** and **I**–**K**)**.**

EC marker analysis was performed to further characterize the newly formed vessels. VEGFR2, a marker associated with active angiogenesis (23), was expressed in all capillary ECs and strongly upregulated in those microvessels that sprouted into the border zone (Figure 5A). It was absent from endocardium (Figure 4E and Figure 5, A and D) and quiescent vessels, such as the large coronary arteries (Figure 5B), as reported previously (24), although there was weak expression of VEGFR2 in some venous ECs (Figure 5C). The subendocardial vessels, which appeared to arise de novo in the MI heart, were also negative for VEGFR2 (Figure 5, E–G and I–K). Based on VEGFR2 expression, the subendocardial vessels did not appear to arise by sprouting angiogenesis or to derive by remodeling and enlargement of the coronary capillaries. In terms of positive markers of identity, both Emcn (Figures 3–5) and endoglin/CD105 (Figure 5L) were found to be highly upregulated in endocardium and subendocardial vessel ECs, which, added to the above, makes coronary arteries an unlikely source of the de novo vessels. To further confirm this, we examined hearts from connexin-40–EGFP (Cx40-EGFP) mice (25) (Figure 5, M–O). Cx40-EGFP is expressed in gap junctions between ECs and coVSMCs; hence, within the coronary vasculature, EGFP expression was limited to coronary arteries (25) (Figure 5M), and following MI, subendocardial vessels were found to be Cx40-EGFP-ve (Figure 5O), consistent with a nonarterial origin. Note that EGFP expression was also observed within Purkinje fibers (Figure 5N), and these fibers were displaced around vessels formed below the endocardial surface in hearts after MI (Figure 5O). Thus, our marker analyses revealed a closer similarity in profile of the endocardium/subendocardial vessels with venous, rather than arterial, ECs, in terms of Emcn, endoglin, and Cx40 expression; the one notable difference being that venous ECs weakly expressed VEGFR2. The basis for distinguishing endocardium from venous ECs came from reexamination of the *Pdgfb*CreERT2 × R26R-EYFP reporter line, in which all coronary ECs, including those of veins, were labeled with high efficiency, whereas 100% endocardial ECs evaded labeling (Figure 2, B and C, and Figure 5, P–R). Examination of subendocardial vessels after MI (Figure 5, P–R) revealed constituent ECs to be entirely negative for EYFP, confirming their derivation from an EC type, or vascular progenitor, that did not express *Pdgfb*/EYFP prior to MI. The distinct marker profile of subendocardial vessels after MI was shared with the endocardium and not with ECs of capillaries, veins, or arteries (Supplemental Table 1), consistent with, and suggestive of, their derivation from the endocardium. Taken together, our findings demonstrate enhanced endocardial trabeculation and ensuing compaction after MI, a direct recapitulation of the perinatal process, which may generate new coronary vessels, as an intrinsic mechanism for neovascularization of the ischemic myocardium.

### Sprouting of coronary sinus veins — reactivation of the adult “sinus venosus.”

**Figure 6:**
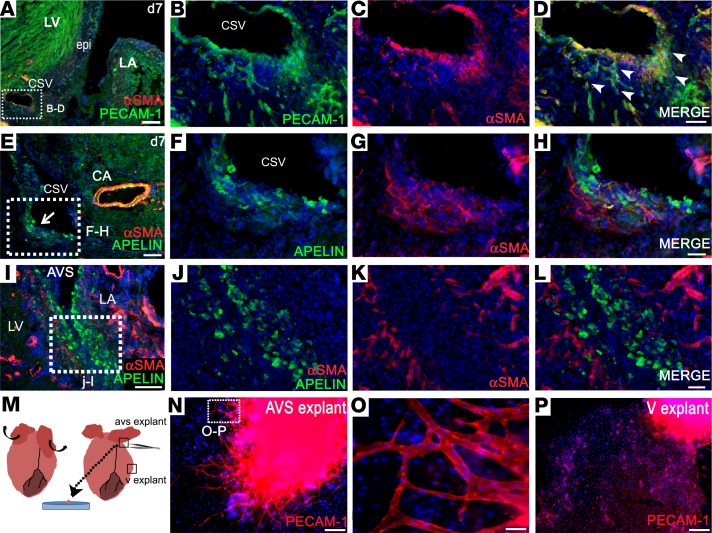
The coronary sinus contributes angiogenic sprouts after myocardial infarction, recapitulating the developmental role of the sinus venosus. Immunostaining on transverse sections of day 7 hearts after MI reveals overt sprouting from the coronary sinus vein (**A**, enlarged in **B**–**D**; sprouting capillaries indicated by arrowheads). Apelin^+^ cells, a marker of sinus venosus in the embryo, comigrate with the PECAM1^+^ cells (**E**, enlarged in **F**–**H**), a smaller proportion of which also coexpress αSMA (**G** and **H**). A gradient of apelin^+^ cells was seen, diminishing in number from the atrioventricular sulcus (**I**, enlarged in **J**–**L**), tracking along the epicardium. In cultures 2 days after MI (**M**), atrioventricular explants produced PECAM1^+^ vascular sprouts by day 7, following outgrowth of epicardial cells at 3–4 days (**N**, enlarged in **O**). In contrast, ventricular explants resulted in outgrowth of epicardial cells but no vascular sprouts, only isolated PECAM1^+^ cells (**P**). Representative immunostaining of *n* = 15 day 7 MI hearts (4 sham); *n* = 4 day 2 MI explant cultures (from each heart, 1 right AVS, 1 left AVS, and 4 ventricular explants were plated in separate wells). LV, left ventricle; LA, left atrium; epi, epicardium; CSV, coronary sinus vein; CA, coronary artery; AVS, atrioventricular sulcus; V, ventricular. Scale bars: 200 μm (**A**); 100 μm (**E**, **I**, **N**, and **P**); 50 μm (**B**–**D**, **F**–**H**, and **J**–**L**); 20 μm (**O**).

Upon systematically assessing all regions of the heart for neovessel formation following injury, we frequently observed sprouting from the coronary sinus vein within the left atrioventricular sulcus, with an apparent breakdown of the vessel wall and extensive outgrowth and branching of ECs (12 of 15 hearts at day 7; representative example in Figure 6, A–D; never observed in *n* = 4 day 7 sham hearts). The emergence of PECAM1^+^ ECs, coexpressing αSMA, a mesenchymal marker, suggested EndMT or partial dedifferentiation (Figure 6, A–D). Overt angiogenic sprouting was never detected from any of the main coronary arteries (e.g., Figure 6E) or from other veins (e.g., Supplemental Figure 1C), not even those in which rare proliferative ECs were observed (Supplemental Figure 1B). Also noteworthy was the expression of apelin, the marker used to trace sinus venosus–derived ECs in the developing heart in small progenitor-like cells, which emerged from the same vessel (Figure 6, E–H) within the atrioventricular sulcus. Apelin^+^ cells were observed to track along the epicardium on both the atrial and ventricular sides of the left atrioventricular sulcus (Figure 6, I–L). Sprouting of veins in this region coincided with an area of precocious epicardial expansion (Figure 6A). To investigate this further, we established atrioventricular sulcus explant cultures, which contained the epicardial layer, from hearts at day 2 after MI. Epicardium-derived cells (EPDCs) emerged from these explants after 3–4 days, and vascular sprouts, consisting of PECAM1^+^ ECs, were apparent after 6–7 days in culture (Figure 6, M–O; sprouts were detected in 3 of 4 left atrioventricular explants and 0 of 4 right explants). Explants from ventricular regions (*n* = 16 explant cultures from 4 MI hearts) consistently produced an outgrowth of epicardial cells but never vascular structures, only a small number of isolated PECAM1^+^ve cells (Figure 6P). Thus, ex vivo data suggested that the coronary sinus is a source of angiogenic, migratory ECs, consistent with histological analyses.

### The reactivated epicardium supports new vessel formation but with minimal cellular contribution.

**Figure 7:**
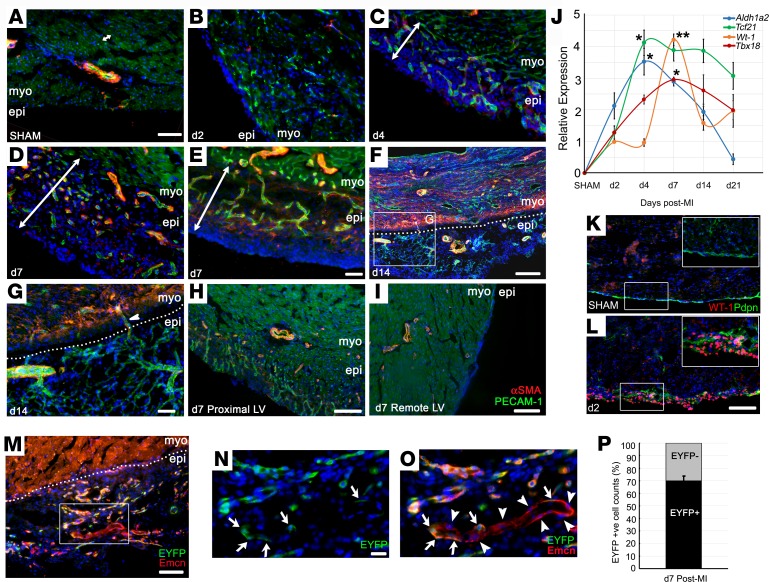
Growth of a vascular network after myocardial infarction within the reactivated epicardium. Shown by immunostaining, the single-cell layer epicardium of the noninjured mouse heart (**A**) expands rapidly in response to injury, from day 2 after MI (**B**), continuing to day 14 (**C**–**F**). A network of capillaries arises within the activated epicardium between day 4 and day 7 (PECAM1^+^ endothelial cells in green; αSMA^+^ vascular smooth muscle cells in red (**C**–**E**). Epicardial capillaries remodel to form arterioles (**F** and **G**), acquiring smooth muscle support (**D**–**H**) and connecting with the underlying coronary vasculature (arrowhead in **G**). Maximal epicardial activity was observed in the proximity of the infarct (**H**), with only modest thickening over remote regions of the LV (**I**). Representative of 24 hours: *n* = 5 (2 sham); 2 days: *n* = 6 (3 sham); 4 days: *n* = 5 (2 sham); 7 days: *n* = 15 (4 sham); 14 days: *n* = 5 (2 sham). qRT-PCR confirmed the reexpression of fetal epicardial genes, *Wt1*, *Tbx18*, *Tcf21*, and *Aldh1a2* (**J**, fold change relative to day 2 MI; *n* = 4 separate animals per time point; mean ± SEM; 2-tailed Kruskal-Wallis nonparametric test with Dunn’s post-hoc test for multiple comparisons; *P ≤ 0.05, ***P ≤ 0.001). WT-1 reactivation in the epicardium, comparing sham (**K**) and day 2 after MI (**L**) hearts by immunostaining (boxes in **K** and **L** correspond to enlarged insets). *Pdgfb*CreERT2; R26R-EYFP pulse-labeling experiments (**M**; box enlarged in **N** and **O**) indicate that 30.1% ± 3.8% of endothelial cells within the expanded epicardium derived from nonendothelial progenitors or endocardium (arrows in **N** and **O** indicate EYFP^+^ cells; arrowheads indicate EYFP^–^ cells; quantified in **P**). LV, left ventricle; epi, epicardium; myo, myocardium. Scale bars: 50 μm (**A**–**E**, **G**, and **M**); 100 μm (**H**, **K**, and **L**); 200 μm (**F** and **I**); 20 μm (**N** and **O**).

**Figure 8:**
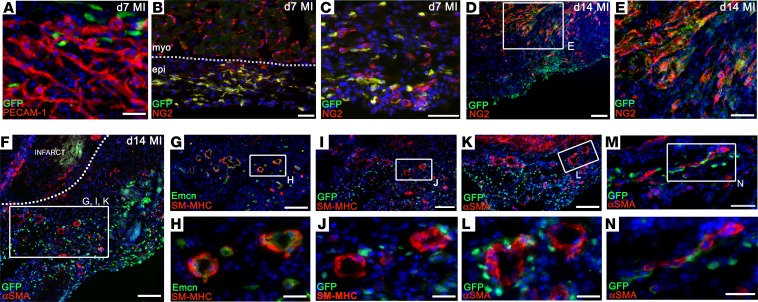
The epicardial lineage contributes pericytes, but not endothelial or smooth muscle cells, after myocardial infarction. *Wt1* lineage tracing (*Wt1*^CreERT2/+^; R26R^EYFP^) with immunostaining revealed no contribution to PECAM1^+^ endothelial cells after infarction (**A**) but revealed extensive contribution of NG2^+^ pericytes by day 7 (**B** and **C**), some of which incorporated into infarct/border zone vessels by day 14 (**D**, enlarged in **E**). No contribution to αSMA^+^/SM-MHC^+^ smooth muscle cells was detected at any stage, shown at day 14, when nonlabeled smooth muscle was abundant in the epicardial vasculature (**F**, equivalent regions in **I**–**N**). EYFP-labeled epicardial cells (GFP^+^) migrate from the epicardium toward the infarct (**F**). Arterioles within the expanded epicardium express the venous marker Emcn, yet they are supported by SM-MHC^+^ cells (**G**, enlarged in **H**). Epicardial lineage cells (GFP^+^) do not contribute endothelial or smooth muscle cells (SM-MHC^+^; **I**, enlarged in **J**; same arterioles shown in **G** and **H**; αSMA^+^; **K**, enlarged in **L**; **M**, enlarged in **N**), although they closely associate with newly formed vessels (**J**, **L**, and **N**). Representative of *n* = 8 hearts at day 7 and *n* = 6 hearts at day 14. epi, epicardium; myo, myocardium. Scale bars: 50 μm (**A**–**C**, **E**, and **M**); 100 μm (**D**, **F**, **G**, **I**, and **K**); 20 μm (**H**, **J**, **L**, and **N**).

Finally, we considered the epicardium as a putative vascular source after MI, given its embryonic contribution and paracrine function to promote coronary vessel development (9). In the uninfarcted mouse heart, the single-cell layer epicardium was morphologically inconspicuous (Figure 7A) and quiescent, with virtually no expression of the canonical epicardial markers (Figure 7, J and K). Within 2 days after injury, a clear thickening of the epicardial layer was observed (3–4 cell layers, Figure 7B), which expanded rapidly over the first 14 days; it was typically 50–100 μm at day 4 (Figure 7C) and 100–200 μm at day 7 to day 14 (Figure 7, D–F), extending both radially and circumferentially, to overlie the infarcted myocardium. Once reactivated, epicardial expansion results, in part, from an increase in proliferation, as evident by the increased incidence of Ki67^+^ cells (Supplemental Figure 2), although we cannot exclude infiltration from other sources, as previously documented (26). Maximal epicardial activity was observed proximal to the infarct (Figure 7H), with only modest thickening over remote regions of the LV (Figure 7I). Epicardial thickness typically peaked at day 14, with a degree of variability dependent on the extent of injury, and gradually declined thereafter (data not shown). qPCR confirmed significant reexpression of fetal epicardial genes, *Wt1*, *Tbx18*, *Tcf21*, and *Aldh1a2*, over a time course, consistent with the histology (Figure 7J). Immunofluorescence on transverse sections showed upregulation of Wilms’ tumor-1 (WT-1) and podoplanin, a mucin-type transmembrane glycoprotein required for epicardial EMT and migration for coronary vessel development (27) (Figure 7, K and L). Concurrent with expansion, a network of capillaries arose within the activated epicardium between day 4 and day 7 (Figure 7, C–E); these progressively remodeled to give rise to a vascular tree and arterioles (Figure 7, F and G), supported by coVSMCs from day 7 (Figure 7, D–H), with connections to the coronary vasculature of the underlying myocardium (Figure 7G). While *Pdgfb*CreERT2 × R26R-EYFP pulse labeling indicated that 69.9% ± 3.8% of ECs within the expanded epicardium derived from preexisting endothelium (EYFP^+^; Figure 7, M–O), a considerable proportion (30.1% ± 3.8%) were from an unlabeled source (Figure 7, N–P). Their putative contribution from epicardial precursors was, however, excluded by fate mapping with an epicardial lineage trace (Wt1CreERT2/^+^; R26R-EYFP/^+^, refs. 20, 28, induced with tamoxifen 48 hours prior to, and upon, LAD ligation, as in ref. 29). We failed to detect a contribution of *Wt1^+^* EPDCs to ECs (Figure 8A), consistent with minimal embryonic contribution from this epicardial subcompartment (9). In contrast, we observed contribution of epicardium-derived NG2^+^ pericytes by day 7 after MI (Figure 8, B and C), some of which incorporated into vessels of the infarct border zone by day 14 (Figure 8, D and E). However, we failed to detect *Wt1* lineage-traced coVSMCs at any stage, even as late as day 21. coVSMCs were present to support newly formed arterioles in the epicardium from day 7 (Figure 7D), which matured further by day 14 (Figure 7F and Figure 8, F–L) (29). These arterioles were unusual, in that they consisted of ECs expressing Emcn, a marker predominantly associated with venous ECs and supported by mural cells expressing SM-MHC, a marker of mature coVSMCs, normally associated with arteries (Figure 8, G and H). Lineage-traced EYFP^+^ EPDCs did not contribute to the SM-MHC– or the αSMA-expressing coVSMCs, even by day 14 (Figure 8, I–L), although they closely associated with these cells (Figure 8, J and L–N).

### Tβ4 recapitulates its developmental role to promote coronary vessel formation after MI.

**Figure 9:**
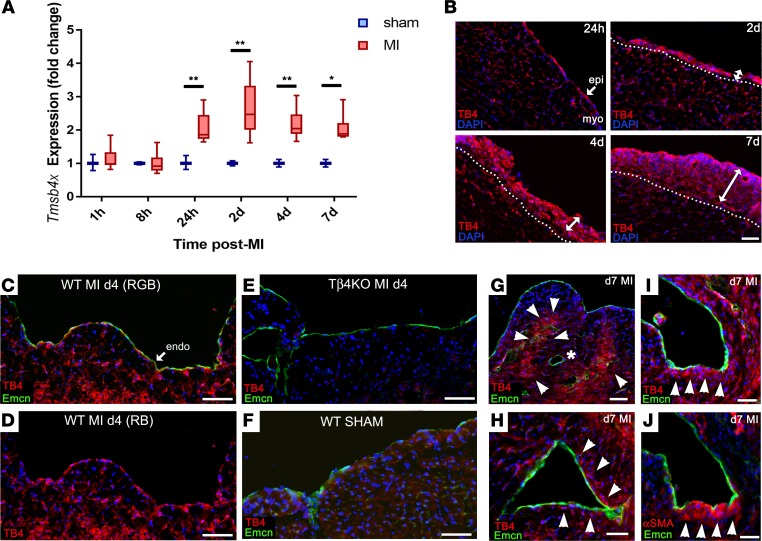
Thymosin β4 expression after myocardial infarction. qPCR shows that *Tmsb4x*, the gene encoding Tβ4, is endogenously upregulated in the heart within 24 hours of infarction (**A**; *n* = 3 separate hearts per time point; box-and-whisker plots show mean ± minimum/maximum; 2-tailed Kruskal-Wallis nonparametric test with Dunn’s post-hoc test for multiple comparisons; *P ≤ 0.05, **P ≤ 0.01. ). By immunofluorescence, Tβ4 levels were highest in the expanding epicardium and upregulated in capillaries (**B**). Tβ4 levels in endocardial cells peaked at day 4 (**C** and **D**). Staining on Tβ4KO sections confirms KO of Tβ4 and antibody specificity (**E**). Upregulation can be appreciated when compared with a sham heart (**F**). By day 7, Tβ4 levels were reduced in endocardium (**G**), but elevated in cardiomyocytes surrounding remodeling subendocardial vessels (arrowheads, **H**) and in cells underlying the endocardium (**I**), many of which coexpressed αSMA (**J**; indicated by arrowheads). Of note, fully formed vessels without associated delaminating cells, indicated by the asterisk in **G**, contained the lowest Tβ4 levels. Representative of *n* = 4 WT day 4 hearts; *n* = 8 WT day 7 hearts and *n* = 8 KO day 7 hearts. MI, myocardial infarction; epi, epicardium; myo, myocardium; endo, endocardium; RGB, red/green/blue channels; RB, red/blue channels. Scale bars: 50 μm (**B**–**G**); 20 μm (**H**–**J**).

Given the roles of Tβ4 in embryonic epicardial migration (17), vascular development (18), and adult epicardial reactivation (29, 30), we sought to determine whether endogenous Tβ4 participates in the intrinsic neovascularization responses after MI. We observed induction of *Tmsb4x*, the gene encoding Tβ4, in the heart within 24 hours of MI (Figure 9A). Immunofluorescence showed Tβ4 levels to be highest in the expanding epicardium (Figure 9B) and also upregulated in capillary ECs (Figure 9B, compared with sham, Figure 9F; Tβ4KO hearts, Figure 9E, confirm antibody specificity). Tβ4 levels in endocardial cells peaked at day 4 (Figure 9, C and D) and decreased by day 7 (Figure 9G), at which stage, Tβ4 accumulated in cardiomyocytes surrounding remodeling subendocardial vessels (Figure 9H) and in cells that appeared to delaminate from the endocardium (Figure 9I), many of which coexpressed αSMA (Figure 9J).

**Figure 10:**
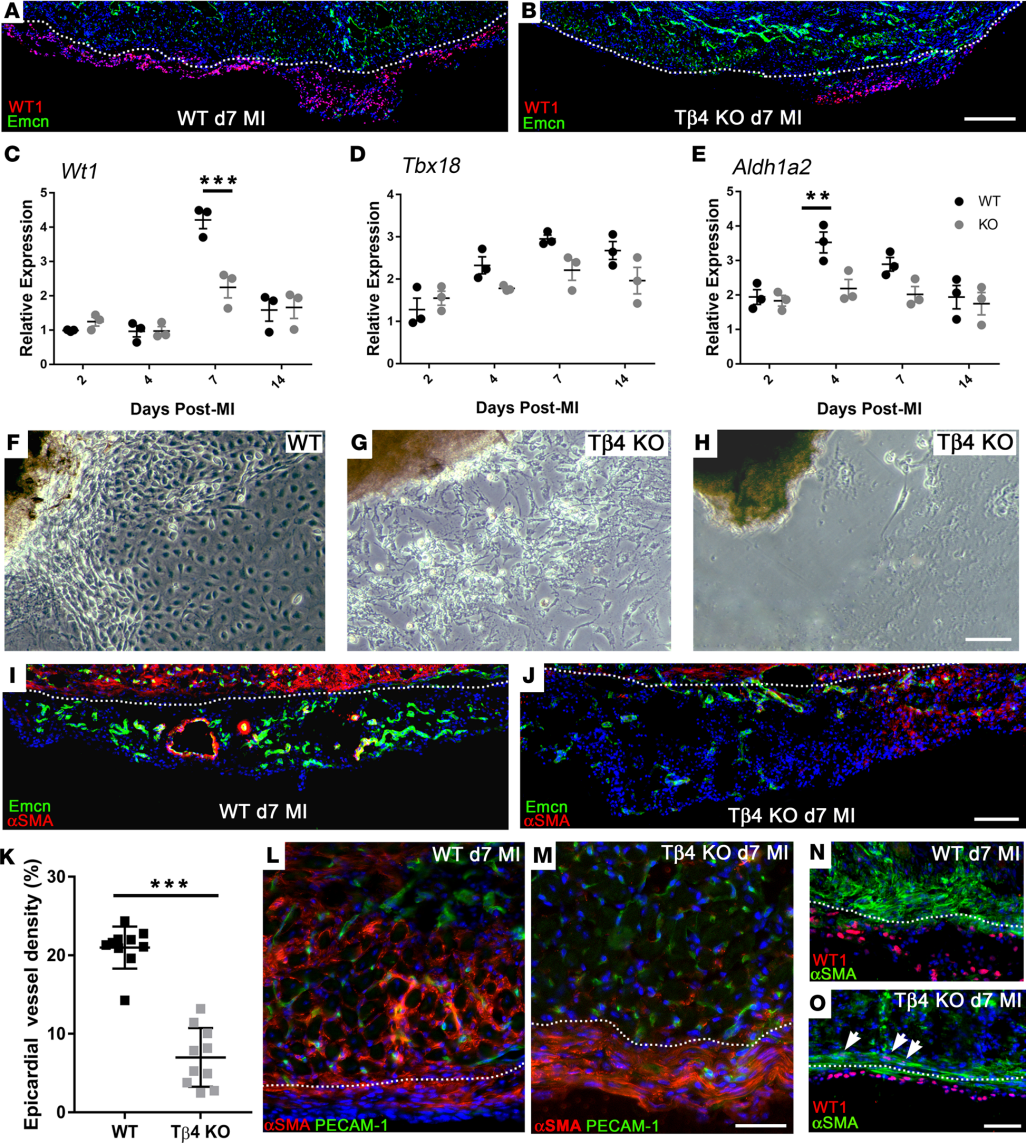
Thymosin β4 is required for epicardial activation, epithelial-to-mesenchymal transition, and expansion of the epicardial neovascular network. Epicardial activation following myocardial infarction was diminished in Tβ4KO hearts (**A** and **B**). By immunostaining, large regions of WT epicardium contained WT-1^+^ cells by day 7 (**A**), compared with only small regions in Tβ4KO hearts (**B**). This was reflected in whole-heart qRT-PCR, with reduced expression of *Wt1* (**C**)*,* *Tbx18* (**D**) and *Aldh1a2* (**E**) by 2-way ANOVA (*n* = 3 separate hearts per time point, mean ± SEM indicated). Post-hoc tests with Bonferroni correction confirmed significant reduction of *Wt1* at day 7 and *Aldh1a2* at day 4. **P ≤ 0.01, ***P < 0.001. Defects in epicardial mobilization were confirmed in explant culture: WT explants outgrew within 7 days (**F**), whereas KO explants produced fewer epicardial cells, and these failed to spread and form an epithelial layer (**G**; *n* = 3 of 8 cultures); moreover, several failed to outgrow altogether (**H**; *n* = 5 of 8 cultures). Coincident with expansion, a smooth muscle–lined vascular network extended throughout the WT epicardium (**I**). Even selecting for regions of greatest epicardial expansion in Tβ4KO hearts, vascular growth was severely diminished and notably lacked smooth muscle support (**J**). Quantification of epicardial vessel density at day 7 confirmed a significant reduction in KO, compared with WT (**K**). Delaminating αSMA^+^ cells invaded the myocardium of WT hearts (**L**), while KO cells failed to migrate inward (**M**). WT cells extended actin cytoskeleton for migration and downregulated WT-1 (**N**); in contrast, Tβ4KO cells remained spindle shaped, failed to orientate for invasion, and retained WT-1 expression (arrowheads, **O**), suggesting incomplete mesenchymal transition. Dotted lines indicate the epicardial-myocardial boundary. Sections are representative of *n* = 10 hearts per genotype and *n* = 10 of each quantified in **K**; each data point represents a separate animal; 2-tailed *t* test; ***P < 0.001. Scale bars: 500 μm (**A** and **B**); 50 μm (**F**–**H**, **L**, and **M**); 200 μm (**I** and **J**); 100 μm (**N** and **O**).

To explore the role(s) of Tβ4 in remodeling of the heart, we performed MI in previously described global Tβ4KO mice (18) and focused on the processes putatively altered by loss of Tβ4, specifically those involving, those involving the epicardium and endocardium. The extent of epicardial activation was severely diminished in Tβ4KO hearts; whereas most of the epicardium overlying the infarct was activated in control hearts by day 7 (revealed by WT-1^+^ve cells, Figure 10A), smaller regions of activation were observed in Tβ4KO hearts (Figure 10B). This was reflected in whole-heart qRT-PCR, with significantly reduced expression at day 7 of *Wt1* (Figure 10C) and *Aldh1a2* (Figure 10E). *Tbx18* was consistently reduced from day 4 to day 14 after MI and statistically significant between genotypes overall (*P* = 0.0103; 2-way ANOVA); however, the reduction did not reach significance for any given time point (Figure 10D). We derived explant cultures to further validate epicardial outgrowth potential (Figure 10, F–H). Without appropriate stimulation, for example, with exogenous Tβ4, adult EPDCs do not readily outgrow from uninjured hearts in culture (17); however, MI stimulates epicardium sufficiently to induce outgrowth in culture. From WT hearts (day 2 after MI), epicardial cells with characteristic cobblestone and spindle morphology (31) emerged from explants within 3–7 days (Figure 10F). In contrast, culture of Tβ4KO hearts 2 days after MI revealed spindle cells exclusively, which failed to spread and form contacts like their control counterparts, and these cultures contained a high proportion of dead cells (Figure 10G). Moreover, 5 of 8 Tβ4KO explants failed to outgrow, even by 14 days (Figure 10H), supporting the phenotype of defective epicardial mobilization. Regions of expanded WT epicardium were rich with capillaries and coVSMC-lined arterioles by day 7 after MI (Figure 10I), whereas, even the areas of greatest epicardial expansion in Tβ4KO hearts failed to sustain the same extent of vascular growth (Figure 10J). Fewer capillaries were observed (7.0% ± 3.7% by area, compared with 20.9% ± 2.7% in WT; Figure 10K), and those present were poorly formed and largely lacked coVSMC support (Figure 10J). These defects are analogous to the coronary defects in cardiac-specific Tβ4KO embryos (17), and, consistent with this, we observed a similar trapping of epicardial cells in KO hearts after MI. Whereas WT epicardial cells (αSMA^+^) had mostly delaminated from the epicardium and migrated through the underlying cardiomyocytes toward the infarct (Figure 10L), Tβ4KO cells underwent partial EMT, to express αSMA, but failed to mobilize from the epicardium (Figure 10M). Closer examination suggested failure to adopt a fully migratory phenotype; WT cells extended actin cytoskeleton and migrated in large numbers into the myocardium (Figure 10N), while Tβ4KO cells remained spindle-like in shape, failed to orientate for myocardial invasion, and many retained WT-1 expression (Figure 10O), suggesting an incomplete transition to a mesenchymal state.

**Figure 11:**
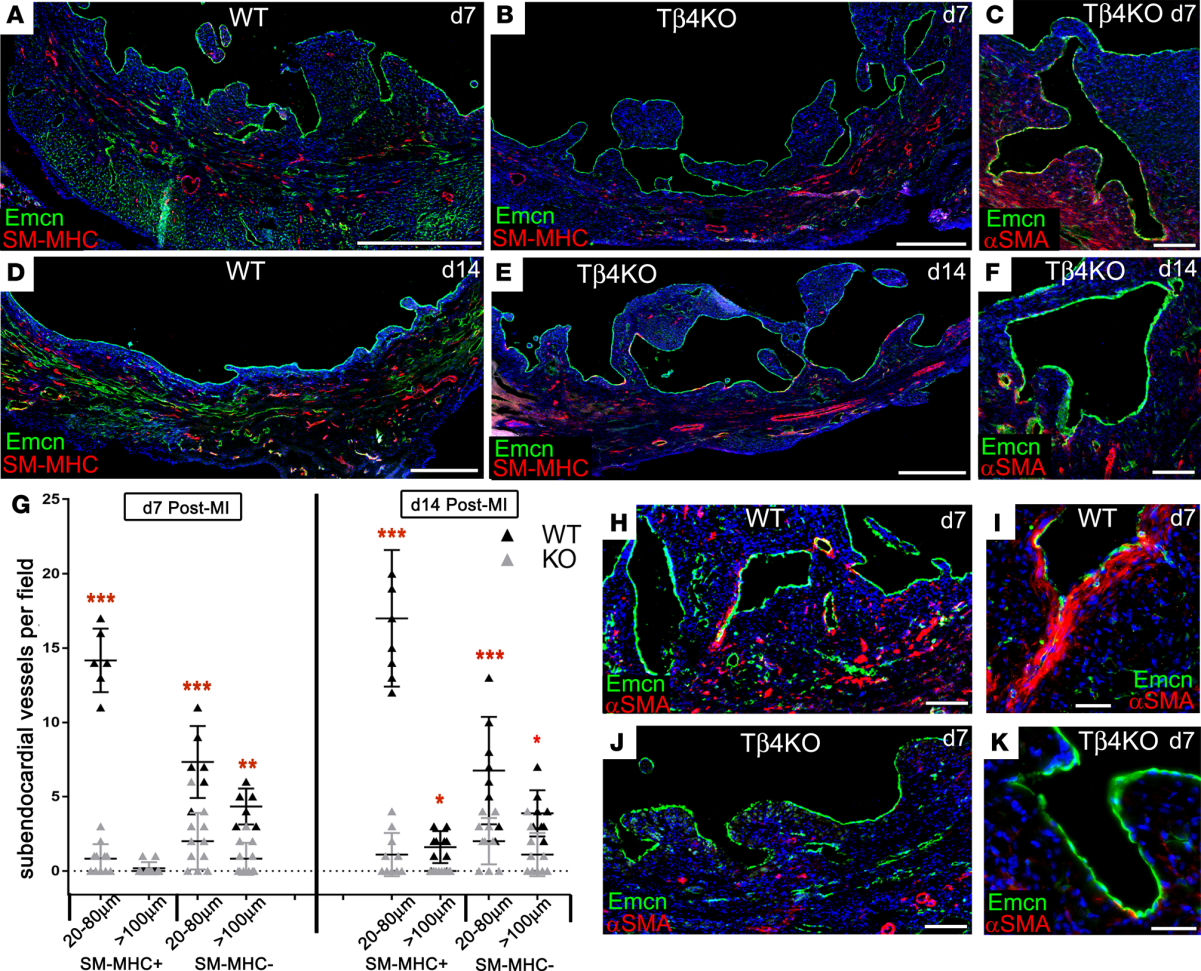
Thymosin β4 is required for trabecular compaction and endocardial contribution of de novo vessels. Compared with WT (**A**), accumulation of trabeculae (**B**) and abnormal lumen morphology (**C**) were evident, by immunofluorescence, in Tβ4KO hearts from day 7 and more apparent by day 14 (**D**–**F**), a stage when compaction is mostly complete in WT (**D**); yet large trabeculae and lumina persisted in Tβ4KO hearts (**E** and **F**), suggesting a failure to undergo compaction remodeling and coalescence of new vessels. These defects manifested as a significant reduction in the incidence of subendocardial vessels formed (representative sections and quantification, **G**) from *n* = 10 hearts per genotype; 1-way ANOVA with Bonferroni correction for multiple comparisons; *P ≤ 0.05, **P ≤ 0.01, ***P < 0.001. This was associated with a striking reduction in αSMA^+^ cells in close proximity to Emcn^+^ endocardial cells (**H**–**K**). Scale bars: 1 mm (**A**, **B**, **D**, and **E**); 100 μm (**C**, **F**, **H**, and **J**); 20 μm (**I** and **K**)

We next assessed the endocardial phenotype of Tβ4KO mice after MI. No defects were apparent prior to day 4, with a comparable extent of induced trabeculation in Tβ4KO hearts (data not shown). However, compared with WT (Figure 11A), we observed, from day 7 onward, a greater accumulation of trabeculae (Figure 11B) and abnormal lumen morphology in the forming subendocardial vessels of Tβ4KO hearts (Figure 11C), suggesting failed compaction remodeling and coalescence of new vessels. Whereas compaction in WT hearts was complete by day 14 (Figure 11D), multiple large trabeculae and lumina, some >2 mm in diameter, persisted in Tβ4KO endocardium at day 14 (Figure 11E). Failed compaction coincided with a lack of supporting coVSMCs (Figure 11F) and a significant reduction in the numbers of subendocardial vessels after MI (Figure 11G). Closer examination of forming lumina showed fewer αSMA^+^ cells underlying the endocardium (Figure 11, H–K), consistent with a possible EndMT defect. Focusing on regions at the base of trabeculae, where αSMA^+^ cells were detected (Supplemental Figure 4A), WT Emcn^+^ endocardial cells strongly expressed nuclear Snai1 at day 4 (Supplemental Figure 4B). By day 7, cells that remained on the endocardial surface, but with downregulated Emcn, expressed αSMA and nuclear Snai1, albeit more weakly than at day 4 (Supplemental Figure 4C). When examining equivalent regions in Tβ4KO trabeculae (Supplemental Figure 4D), the reduction in migratory αSMA^+^ cells was accompanied by a reduced percentage of Snai1^+^ endocardial cells (Supplemental Figure 4, E–G). Very few endocardial cells were positive for Snai1, and, in most of these, the transcription factor was not nuclear localized (Supplemental Figure 4E); also of note, some endocardial cells displayed an abnormal, rounded morphology, and these simultaneously expressed αSMA and Emcn (Supplemental Figure 4F). The possible EndMT defects suggested by these data were supported by qRT-PCR showing reduced levels of both *Snai1* and *Snai2* (Supplemental Figure 4, H and I).

**Figure 12:**
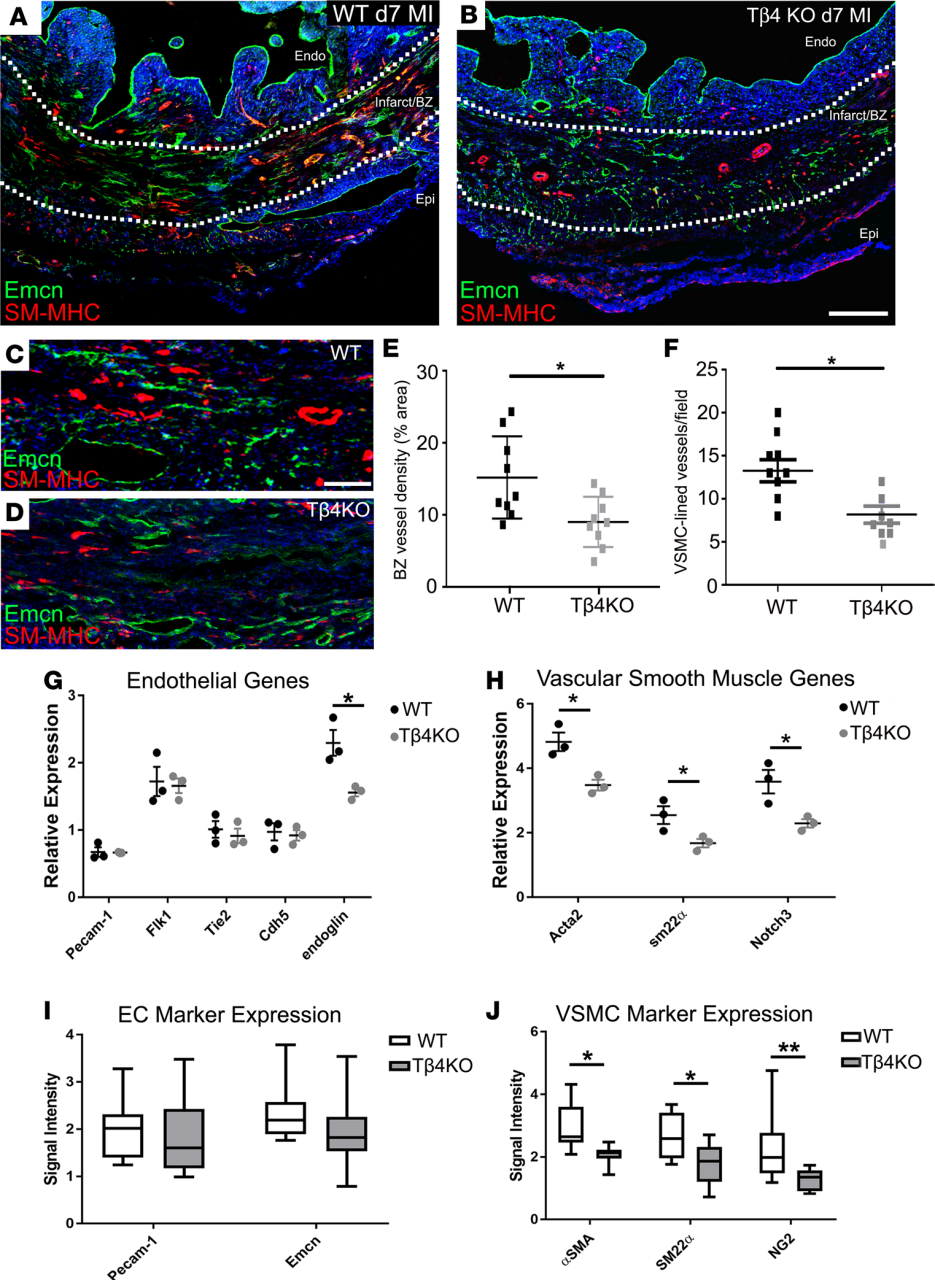
Neovascularization is diminished in the infarct border zone of thymosin β4 KO hearts. By immunofluorescence, vascular density within the border zone was significantly reduced in Tβ4KO hearts, compared with WT (**A**–**D**, quantified in **E**), and vessels appeared less mature (**B**, compared with **A**). Fewer vessels acquired smooth muscle support (**D**, compared with **C**, quantified in **F**). By qRT-PCR, endothelial cell markers were not significantly reduced, with the exception of endoglin (**G**), whereas smooth muscle markers, *Acta2*, *Sm22a* and *Notch3*, were all significantly reduced in Tβ4KO hearts (**H**). These differences were reflected in quantification of immunofluorescence signal intensity, showing no significant reduction of endothelial markers (**I**) but a significant reduction of smooth muscle markers (**J**). Sections are representative of *n* = 9 hearts per genotype and *n* = 9 quantified. BZ, border zone; endo, endocardium; epi, epicardium. Scale bars: 500 μm (**A** and **B**); 50 μm (**C** and **D**). Statistical analyses (**E** and **F**): Mann-Whitney test (2-tailed); (**G**–**J**) 2-way ANOVA with Bonferroni correction for multiple comparisons; scatter plots: each data point represents a separate animal with mean ± SEM; box-and-whisker plots show mean ± minimum/maximum; *P ≤ 0.05, **P ≤ 0.01.

Finally, we assessed the vasculature within the border zone immediately surrounding the infarct. Vascular density in this region was significantly reduced in Tβ4KO hearts, compared with WT hearts (Figure 12, A–E), and vessels appeared less mature. Notably, fewer vessels acquired coVSMC support (Figure 12, C, D, and F). By qRT-PCR, EC markers were not significantly reduced, with the exception of endoglin (shown at day 7, Figure 12G), which has specific roles in angiogenesis (32) and coVSMC recruitment (33). Consistent with this and known roles of Tβ4 in coVSMC differentiation, levels of coVSMC markers, *Acta2*, *Sm22a*, and *Notch3*, were significantly reduced in Tβ4KO hearts at day 7 (Figure 12H). These differences were reflected at the protein level, with no reduction of EC markers, yet a significant reduction of coVSMC markers (Figure 12, I and J). Taken together, these data suggest a requirement for Tβ4 in angiogenesis, remodeling, and coVSMC recruitment to vessels in the infarct border zone.

## Discussion

Collectively, our study provides descriptive evidence to support the apparent recapitulation of developmental mechanisms, with roles for all three recognized embryonic EC sources, toward neovascularization of the ischemic adult heart. The sinus venosus is a transient developmental structure, which does not exist as such in the adult; however, the coronary sinus veins act as an equivalent source. Similarly, an endocardial contribution would be expected to cease with compaction in the early postnatal period and the epicardium loses natural capacity to migrate, differentiate, and deliver potent paracrine factors to the myocardium shortly after birth (34). However, in the context of myocardial injury, these quiescent lineages in the adult respond to endogenous injury cues to instigate a reparative program and revascularization of the ischemic heart.

By prelabeling coronary ECs prior to infarction, we reveal a contribution of noncoronary ECs to enhance perfusion of infarcted myocardium after MI. The majority of unlabeled ECs were identified within the remodeled capillary network that sprouts into the infarct border zone, within the thickened epicardium and in subendocardial vessels. Previous inducible endothelial lineage traces (15), based on *Cdh5*CreER, *Apln*CreER, and *Fabp4*CreER, captured 100% of PECAM1^+^ ECs, including those of the endocardium, and reported 100% labeling of ECs after MI. We avoided labeling endocardial ECs in the *Pdgfb*CreER trace and report 26.4% EYFP^–^ ECs in the border zone and 30.1% EYFP^–^ ECs within the epicardial vasculature. Border zone and epicardial capillaries were frequently heterogeneous, comprising both EYFP^+^ and EYFP^–^ ECs, whereas the larger subendocardial vessels were entirely devoid of EYFP. The appearance of subendocardial vessels, coinciding with closure of endocardial lumina and compaction of injury-induced trabeculae, along with their identical molecular marker profile, is consistent with an endocardial origin, but definitive proof awaits the identification of an endocardial-specific marker for lineage tracing in the adult. The coincident phenotypes of failed compaction and absence of subendocardial vessels in Tβ4KO hearts further supports an endocardial contribution. The source(s) of the heterogeneous border zone and epicardial capillaries is more ambiguous. While the coronary sinus, upstream of the infarct in the left atrioventricular sulcus, was the only site of overt sprouting, with directional expansion of vessels tracking progressively toward the infarct via the epicardium, the absence of a suitable lineage-specific marker limits our ability to definitively demonstrate its contribution to the EYFP^+^ capillaries in the active epicardium. The EYFP^–^ ECs, which incorporated into the mixed origin capillaries, may be endocardial in origin or recruited from a *Pdgfb*^–^ vascular progenitor population.

The plasticity of the adult endocardium was recently described, with the formation of vascular structures, resembling “flowers,” on the luminal endocardial surface after MI. These structures associated with subendocardial coVSMCs and were connected, via stalk-like structures, to the underlying coronary vessels (24). We describe herein the formation of larger “conduit vessels,” with a caliber in the range of large arterioles to small arteries, in the subendocardium following infarct. Although endocardial flowers and subendocardial vessels develop and mature over a similar 14-day window after MI, they are dissimilar in structure, molecular identity, and mechanism of formation. The constituent ECs of endocardial flowers were Emcn^–^, endoglin^–^, VEGFR2^+^, and, by day 7, Cx40^+^, whereas subendocardial vessels express the equivalent profile of the endocardium itself (Emcn^+^, endoglin^+^, VEGFR2^–^, and Cx40^–^), in keeping with the notion of inward remodeling of the luminal surface and coalescence as a plausible mechanism for their derivation. In line with expression of VEGFR2, an angiogenic marker, the ECs and αSMA^+^-supporting cells of flowers were found to express proliferation markers Ki67 and PHH3 (24). These distinct mechanisms reflect recapitulation of two discrete processes of endocardial vessel contribution in development: the embryonic complement, which is shown to derive from myocardial VEGF-A/endocardial VEGFR2 signaling to induce angiogenesis (8), manifesting in the adult as an endocardial flower formation, versus the larger arterioles, which derive from induced trabeculation and subsequent compaction, as proposed to account for the perinatal expansion of the coronary vasculature, and associated with minimal proliferation of endocardial cells and no visible sprouting. The notion that the adult heart should revert to its former hypertrabeculated state and repeat the process of compaction to facilitate neovascularization of the ischemic tissue is, to our knowledge, entirely novel; yet, it is both logical, given the presence of a convenient reservoir of blood in the nearby ventricular lumen, and has precedence in humans. For example, induced hypertrabeculation has been reported in response to increased preload, presenting as noncompaction in some elite athletes (35) and pregnant women (36).

Expression of endoglin, VEGFR2, and proliferation markers in rare venous, but not arterial, ECs suggests a greater potential of veins for angiogenic sprouting in response to injury. This is consistent with the mechanism of vascular plexus remodeling and derivation of arteries by venous EC proliferation and sprouting shown to occur during development of the mouse retina and regeneration of the fin in zebrafish (37). It is also the basis of embryonic coronary vessel outgrowth from the sinus venosus (7); sprouting venous ECs of the sinus venosus dedifferentiate, migrate over the epicardial surface, and reacquire venous or arterial identity, depending on their eventual location within the myocardium. It remains unclear why the coronary sinus, rather than more proximal coronary veins, undergoes angiogenic sprouting following MI. A distinct molecular identity within the coronary sinus may impose an embryonic-like potential following injury-induced reactivation, suggesting a retained memory of its developmental role as the emergent sinus venosus. An alternative explanation may be the precocious and robust epicardial activation in the atrioventricular region, despite its distance upstream of the occluded artery, which may serve to provide an inductive cue, analogous to epicardial VEGF-C stimulation of sinus venosus sprouting in development (9). It will be interesting to determine whether the stimulus for inducing a response in the coronary sinus may be a long-range molecular signal or an alteration in hemodynamic forces, which is known to promote angiogenesis (38).

Epicardial involvement in neovascularization is more difficult to define. Epicardial activation is required for regeneration of the zebrafish heart (39), and lineage-tracing experiments demonstrated epicardial contribution to perivascular cells in this species (40). Reexpression of embryonic epicardial genes occurs even in the nonregenerating mouse heart after MI (29, 41, 42). We describe the assembly of a de novo capillary network within the expanded epicardium after MI, which remodels and becomes arterialized. Despite its formation within activated epicardium, we found no direct cellular contribution of *Wt1^+^* cells to the ECs or coVSMCs of the vascular network. Embryonic contribution of coVSMCs from the *Wt1*/*Tbx18*-expressing lineages has been reported (28, 43–45), with pericytes as the epicardium-derived intermediaries that gave rise to coVSMCs (45). We report an epicardial (*Wt1^+^*) contribution to pericytes in the adult heart after MI, but we observed no progression to mature coVSMCs. While the environment after MI may not support the appropriate signaling, such as Jagged1/Notch3, implicated in development (45), to permit pericyte-coVSMC transition, the coVSMCs that surround arterioles in the subepicardium after MI must derive from a non-*Wt1*^+^ lineage. These findings are consistent with previous reports that adult epicardial coVSMC contribution is extremely rare without exogenous stimulation (30, 46, 47), yet the literature perpetually overstates the extent (e.g., refs. 48, 49), either because nonspecific markers, such as αSMA, were used to define coVSMCs or because inducible epicardial-specific lineage tracing was not used in many of the cited studies. Despite the lack of contribution, EYFP^+^ cells were found to closely associate with arteriolar coVSMCs, perhaps contributing adventitial fibroblasts or supporting the coalescence and differentiation of new vessels indirectly, via modulation of ECM or paracrine secretion. We also confirm previous findings of a lack of EC contribution, as seen with lineage traces based on *Wt1* (46, 47), *Gata5* (30) *Tcf21* (*40*), or *Mesothelin* (47). Given that a distinct subcompartment of the proepicardium expressing *Sema3d* and *Scx* contributes the 5%–15% of coronary ECs in development, a degree of adult EC contribution, possibly to the EYFP^–^ epicardial ECs, may have been overlooked; however, as neither *Sema3d* nor *Scx* are expressed specifically in adult epicardium, options for lineage tracing of additional populations are currently limited. An alternative explanation for the origin of the epicardial vascular network may be the outgrowth of venous-derived capillaries from coronary sinus sprouting, stimulated by activated epicardium, essentially as occurs in development. We demonstrate the strong capacity of the epicardium to support growth of a vascular network; epicardial cells secrete potent angiogenic growth factors (50) and provide a suitable ECM to promote expansion of the neovessel network toward the infarcted myocardium (51, 52).

The pleiotropic roles for Tβ4 in neovascularization after MI are consistent with previously demonstrated functions, either in development or disease. The direct parallel of failed epicardial mobilization, leading to defective coronary vasculogenesis, was a feature of cardiac-specific Tβ4 knockdown embryos (17). Transition to a mesenchymal state and migration, a hallmark of mesenchymal cells, are intricately linked. Whether the apparent EMT and EndMT defects, from the epicardium and endocardium, respectively, reflect a requirement for Tβ4 to drive aspects of mesenchymal transformation or for extension of the actin cytoskeleton to enable migration cannot be easily separated. Both are plausible; (a) the persistent expression of WT-1, a regulator of epicardial EMT (53), in αSMA^+^ epicardial cells implies an incomplete EMT, and Tβ4 has been reported to promote EMT for metastasis (54, 55); (b) the earliest identified function for Tβ4 was as an actin monomer-sequestering molecule, implicated in cytoskeletal remodeling (56). Consistent with our findings, exogenous Tβ4 has been shown to promote angiogenesis (57) and to drive VSMC differentiation in vascular development (18, 58) and neovascularization after MI (30, 59).

Understanding the intrinsic mechanisms that aim to reestablish blood flow to infarcted myocardium may reveal novel targets for stimulating cardiac regeneration. With recently acquired insights into the embryonic program and cellular origins of progenitors that establish the coronary vasculature, we are now better placed to interrogate the plasticity and potential of the equivalent populations to contribute in the adult. Our study provides descriptive evidence for the redeployment of the endocardium and coronary sinus to contribute new vessels after MI, with a supporting role for the epicardium to promote the expansion and directional outgrowth of the emergent vascular network toward the infarcted myocardium. These fundamental observations provide the underpinning to enable further mechanistic insight into the pathways that regulate revascularization of the injured heart as a basis for developing novel therapies to drive an enhanced neovascular response.

## Methods

Supplemental Methods are available online.

### Mouse strains.

*Pdgfb*CreERT2 mice (a gift from Marcus Fruttiger, UCL Institute of Ophthalmology, London, United Kingdom) have previously been reported (16) and were crossed with Rosa26R (R26R)-EYFP reporter mice (a gift from Shankar Srinivas, University of Oxford, Oxford, United Kingdom) (20), to generate *Pdgfb*CreERT2; R26R-EYFP mice. Expression of EYFP in ECs was induced by two injections of 2 mg tamoxifen (for ~25 g mouse), 2 days apart, 16 and 14 days prior to MI. Previously described WT1CreERT2/^+^ mice (28) (a gift from William Pu, Boston Children’s Hospital, Harvard Medical School, Boston, Massachusetts, USA) were also crossed onto the R26R-EYFP line and induced with 2 mg tamoxifen 2 days prior to, and on the day of, MI. In the previously described Cx40-EGFP line (25) (a gift from Lucile Miquerol, Aix-Marseille University, Provence, France), EGFP was knocked into the *Gja5* locus. As previously described (18), global *Tmsb4x* KO mice (a gift of Martin Turner, Babraham Institute, Cambridge, United Kingdom) were generated by deleting exon 2 of the *Tmsb4x* gene. All strains were maintained on a C57BL6/J background for more than 20 generations, with the exception of the Cx40-EGFP line, which was maintained on a mixed CD1/129Sv background for more than 13 years.

### Acute MI model — permanent LAD ligation.

Male mice (<35 g, age 10–18 weeks) underwent surgery to induce MI, using aseptic technique. Briefly, the mouse was anesthetized with isoflurane (97% O_2_/2% [vol/vol]) and maintained at 37°C, in the supine position. Respiration was controlled via an endotracheal tube and a ventilator (stroke volume of ~200 μl/min and ~200 strokes/min). MI was induced by permanent ligation of the LAD. Sham-operated animals underwent intubation, thoracotomy, and insertion of the suture trough the LV but no ligation. 0.015 mg/ml Buprenorphine hydrochloride was delivered via intraperitoneal injection 10 minutes before the procedure to provide analgesia (and 24 hours after MI, where required).

Mice were killed by cervical dislocation, and hearts were dissected at 24 hours, day 2, day 4, day 7, day 14, and day 21 after MI. The extent of MI in each heart was initially assessed by immunofluorescence on midventricular transverse cryosections against cardiac troponin T and staining with 300 nmol/μl DAPI. As a predefined exclusion criterion, hearts with <20% or >50% of the LV infarcted were excluded from the study in an attempt to limit variability, which results primarily from the inconsistent placement of the suture along the LAD and variable coronary anatomy in the mouse. The sample sizes indicated throughout reflect the total number analyzed, after exclusion of animals with insufficient or excessive myocardial injury.

For RNA isolation, hearts were snap frozen in liquid nitrogen, after removal of a thin slice for scoring of injury, as above, and processed as described below. For cryosectioning, hearts were fixed in diastole by injection of 1 M KCl into the right atrium, flushed with PBS and perfused with 4% paraformaldehyde in PBS (PFA), prior to a 2 hour fixation in 4% PFA, at room temperature

### Immunofluorescence staining on cryosections.

Full details regarding antibodies used are provided within the Supplemental Methods.

Fixed hearts were equilibrated overnight in 30% sucrose/PBS at 4°C and embedded in OCT Compound (Tissue-Tek). Frozen sections were cut at a thickness of 10 μm, air dried for 10 minutes, and then rinsed in PBS. Sections were permeabilized in 0.5% Triton X-100/PBS for 10 minutes, rinsed in PBS, and blocked for 1–2 hours in 1% BSA, 10% goat serum (or donkey serum when primary antibodies were raised in goat), 0.1% Triton X-100 in PBS (PBST). Incubation with primary antibodies, diluted in blocking buffer, was at 4°C overnight. Sections were washed 5 times for 5 minutes each time in PBST and then incubated in secondary antibody, in blocking buffer, for 1 hour at room temperature. Sections were washed 3 times in PBST and incubated with DAPI/PBS for 5 minutes and rinsed twice in PBS. Slides were mounted with 50% glycerol in PBS and imaged using a Zeiss Axioscope or Leica DM6000 fluorescence microscope.

### Quantification of vascular marker expression following immunofluorescence.

Using ImageJ (NIH), images were thresholded to exclude background fluorescence, and total channel fluorescence was quantified to determine mean fluorescence. Particle analysis was performed to determine the percentage area occupied by fluorescent signal. Since mean pixel intensity is influenced by dark space (background), the mean fluorescence was normalized against percentage area to obtain mean intensity of vascular signal.

### RNA isolation and qPCR.

Sequences of all primers used are provided within the Supplemental Methods.

Whole hearts were homogenized in Trizol reagent (Invitrogen) at a volume of 1 ml/50–100 mg tissue and RNA was extracted, according to the manufacturer’s instructions. Total RNA was quantified using a Nanodrop (Thermo Fisher Scientific). Reverse transcription was performed using 0.5 μl 500 μg/ml random primers and Superscript II Reverse Transcriptase (Invitrogen). qPCR analysis was performed on an ABI 7000 Sequence Detector using SYBR Green (Applied Biosystems). Data were normalized to *Hprt1*, and relative expression was calculated using the ΔΔC_t_ method (60). For epicardial genes, fold change from baseline to day 2 is highly underestimated in our analysis, as epicardial markers are barely expressed in the uninjured adult heart and cycle threshold (C_t_) values from those times points were too high to accurately derive expression level; therefore, fold change is shown relative to day 2 values. The extent of reactivation and marker reexpression is better demonstrated at the protein level by immunofluorescence; however, qPCR is included to quantitate temporal changes between day 2 and day 21.

### Adult epicardial explants.

Culture dishes were precoated with 0.1% gelatin and air dried. Hearts after MI (day 2) were collected in explant medium (a 1:1 mixture of low-glucose Dulbecco’s modified Eagle’s medium and Medium 199 with 15% ES FCS, 1 % Glutamax, and 1% nonessential amino acids; all from Invitrogen) and placed in PBS with 1% penicillin/streptomycin, while the epicardium of each heart was carefully peeled away, taking care to separate atrioventricular and myocardial portions into separate wells of a 6-well plate. Coverslips were placed on top of the tissues and pressed down to encourage adhesion. Explants were air dried at 37°C for 30 minutes and then explant medium was added to cover the sample, which was incubated at 37°C, with daily monitoring

### Statistics.

Power calculations, based on our previous studies determined that a minimum of 5 animals per time point were required to assess the extent of epicardial activation and de novo vessel formation. Predefined exclusion criteria were used to exclude samples in which infarcts were <20% or >50% LV myocardium. Blinding was used for assessment of coronary sinus sprouting, where images were reassessed and scored for sprouting by an independent observer while blinded to treatment (MI vs. sham). Randomization of animals to sham versus MI groups was determined by the surgeon at the time of LAD ligation.

Statistical analyses were performed with GraphPad Prism software. For the quantitative comparison of *Pdgfb*EYFP^+^ECs before/after MI, a 2-tailed unpaired Student’s *t* test was used to determine any significant differences. The requirements for a *t* test were assessed using a Shapiro-Wilk test for normality and an F test to compare the variances. For the comparison of subendocardial vessel density, a 1-way ANOVA with Bonferroni correction was used. Vessel density, marker expression, and qPCR data in Tβ4KO versus WT were analyzed using a Kruskal-Wallis nonparametric test with Dunn’s post-hoc test for multiple correction. *P* ≤ 0.05 was considered significant.

### Study approval.

Mice were housed and maintained in a controlled environment, and all procedures involving the use and care of animals were performed in accordance with the Animals (Scientific Procedures) Act 1986 (Home Office, United Kingdom) and were approved by UCL and the University of Oxford Animal Welfare and Ethical Review Boards.

## Author contributions

KND conducted experiments and analyzed data, with additional data acquired by TMT and SM and surgical assistance from MR. PRR provided intellectual input and edited the manuscript. NS conceived and supervised the study, conducted experiments, analyzed data, and wrote the manuscript.

## Supplementary Material

Supplemental data

## Version 1 11/16/2017

Electronic publication
