# Supplementary material for: Recapitulation of developmental mechanisms to revascularize the ischemic heart
Source: JCI Insight. 2017 Nov 16;2(22):e96800. doi: 10.1172/jci.insight.96800 (PMC5752387; doi:10.1172/jci.insight.96800)
Supplement: Supplemental data [file jciinsight-2-96800-s001.pdf]

ONLINE SUPPLEMENT

Extended Experimental Procedures

Mouse Strains

Mice were housed and maintained in a controlled environment and all procedures involving the use and care of animals were performed in accordance with the Animals (Scientific Procedures) Act 1986, (Home Office, United Kingdom) and approved by UCL and University of Oxford Animal Welfare and Ethical Review Boards. *Pdgfb*CreERT2 mice (gift from Marcus Fruttiger, UCL, London), with insertion of a tamoxifen inducible form of Cre recombinase into the open reading frame of the *Pdgfb* gene, have previously been reported[1] and were crossed with Rosa26R (R26R)-EYFP reporter mice (gift from Shankar Srinivas, University of Oxford[2], to generate *Pdgfb*CreERT2; R26R-EYFP mice. Expression of EYFP in endothelial cells was induced by two injections of 2mg tamoxifen (for ~25g mouse), 2 days apart, 16 and 14 days prior to LAD ligation. Previously described WT1CreERT2/+ mice (gift from William Pu, Boston Children's Hospital, Harvard Medical School[3] contain CreERT2 knocked into the *Wt1* locus. These were also crossed onto the R26R-EYFP line and induced with 2mg tamoxifen 2 days prior to, and on the day of, LAD ligation. In the previously described Cx40-EGFP line[4] (gift from Lucile Miquerol, Aix-Marseille University), EGFP was knocked into the Cx40 (*Gja5*) locus. As previously described[5], global *Tmsb4x* knockout mice (gift of Martin Turner, Babraham Institute, Cambridge) were generated by deleting exon 2 of the *Tmsb4x* gene. All strains were maintained on a C57Bl6/J background for more than 20 generations, with the exception of the Cx40-EGFP line, which was maintained on a mixed CD1/129Sv background for more than 13 years.

Acute Myocardial Infarction Model: Permanent LAD Ligation

Wild type and transgenic male mice (<35g, age 10-18 weeks) underwent ligation of the left anterior descending artery to induce MI, using aseptic technique. Briefly, the mouse was anaesthetised with isoflurane (97% O_2/2% (vol/vol) and maintained at 37°C, in the supine position. Respiration was controlled via an endotracheal tube and a ventilator (stroke volume of ~200µl min-1 and ~200 strokes min-1). MI was induced by permanent ligation of the left descending artery (LAD). Sham-operated animals underwent tracheotomy, thoracotomy and insertion of the suture trough the left ventricle, but no ligation. Buprenorphine hydrochloride (Vetergesic) was delivered as a 0.015 mg ml−1 solution via intraperitoneal injection 10 minutes before the procedure to provide analgesia (and 24h post-MI, where required).

Mice were killed by cervical dislocation and hearts were dissected across a range of time points post MI: 24h (n=5; n=2 sham); d2 (n=6; n=3 sham); d4 (n=5; n=2 sham); d7 (n=15; n=4 sham); d14 (n=5; n=2 sham). Extent of myocardial infarction in each heart was assessed after immunofluorescence on cryosections against cardiac troponin T or myosin light chain 2 (MLC2) and staining with 300nmol/µL 4',6-diamidino-2-phenylindole (DAPI). As a pre-defined exclusion criterion, hearts with <20% or >50% of the LV infarcted were excluded from the study as the injury response is known to vary considerably with extent of injury, variability of which results primarily from the precise placement of the suture along the LAD and variable coronary anatomy in the mouse. The sample sizes indicated throughout reflect the total number analysed, after exclusion of animals with insufficient or excessive myocardial injury.

For RNA isolation, hearts were snap frozen in liquid nitrogen, after removal of a thin slice for scoring of injury, as above, and processed as described below. For cryosectioning, hearts were fixed in diastole by injection of 1M KCl into the right atrium, flushed with PBS and perfused with 4% paraformaldehyde in PBS (PFA), prior to a 2 hour fixation in 4% PFA, at room temperature

Immunofluorescence Staining on Cryosections

Fixed hearts were equilibrated overnight in 30% sucrose in PBS at 4°C, sucrose removed by incubation in 50:50 30% sucrose PBS:OCT and 100% OCT, before embedding in 100% OCT and chilling at -80°C. Frozen heart sections were cut at a thickness of 10 µm, air-dried for 5 minutes and then rinsed in PBS.

Sections were permeabilised in 0.5% Triton-X100/PBS for 5 minutes, rinsed in PBS and blocked for 1-2 hours in 1% BSA, 10% goat serum (or donkey serum when primary antibodies were raised in goat), 0.1% Triton-X100 in PBS). Incubation with primary antibodies, diluted in blocking buffer, was at 4°C overnight. Sections were washed 5-6 times in 0.1% Triton-X100 in PBS (PBST) over a period of 1 hour then incubated in secondary antibody, diluted in blocking buffer, for 1 hour at room temperature. Sections were washed 3 times in PBST and incubated with 300nmol/µL 4',6-diamidino-2-phenylindole (DAPI) in PBS for 5 minutes and rinsed twice in PBS. Slides were mounted with 50% glycerol in PBS and imaging was conducted on a Zeiss fluorescence microscope with Axiovision 4.4 software or a Leica DM6000 fluorescence microscope.

Antibodies for Immunofluorescence

The following primary antibodies were used: Rat anti-PECAM-1, (1:50; BD Pharmingen; Cat. 557355); Cy3 conjugated Mouse anti-αSM actin (1:200; Sigma; Cat. C6198); Chicken anti-GFP (1:1000; Abcam; Cat. ab13970); Rabbit anti-Apelin (1:250; Abcam; Cat. ab59469); Goat anti-CD105 (1:25; R&D Systems; Cat. AF1320); Rabbit anti-WT-1 (1:100; Abcam; Cat. ab89901); Rabbit anti-Vimentin (1:200; Abcam; Cat. ab16700); Rabbit anti-MLC2 (1:150; Abcam; Cat. ab79935); Rabbit anti-Fibronectin (1: 100; Abcam; Cat. ab23750); Rat anti-CD44 (1: 100; Abcam; Cat. ab25340); Rat anti-Endomucin (1:50; Santa Cruz Biotechnology; Cat. sc-53941); Rabbit anti-Snai1 (1:100; Abcam; Cat. Ab180714); Rabbit anti-Thymosin β4; 1:100; Immundiagnostik; Cat. AA9520.2); Rabbit anti-SM-MHC (1: 1000; Abcam; Cat. ab125884); Hamster anti-Podoplanin (1:100; Fitzgerald; Cat. 10R-P155A); Rat anti-Ki67 (1:100; eBioscience; Cat. 14-5698); Rabbit anti-P-HH3 (1:200; Millipore; Cat. 06-570); Rabbit anti-NG2 (1:200; Millipore; AB5320); Mouse anti-α-SA (1:500; Sigma; Cat. A7811); Rabbit anti-CC3 (1:400; Cell Signalling Technology; Cat. 9661); Goat anti-VEGFR2 (10µg/ml; R&D Systems; Cat. AF66). AlexaFluor (488/546/647) secondary antibodies, raised in goat or donkey, were used (Invitrogen) at 1:200. All antibodies have been validated for use in immunofluorescence by 1Degree BIO, with the exception of the apelin, GFP, MLC2, endomucin, SM-MHC and VEGFR2 antibodies, which have been validated by the manufacturer, endorsed by user reviewers and verified in previous publications.

RNA Isolation and qPCR

Whole hearts were collected post sham and LAD ligation operations, homogenized in Trizol reagent (Invitrogen) at a volume of 1 mL/ 50-100 mg tissue and RNA extracted, according to the manufacturer's instructions. Total RNA was quantified using a Nanodrop. Reverse transcription was performed using 0.5µL 500 µg/mL random primers and Superscript II Reverse Transcriptase (Invitrogen) and cDNA was diluted to 4 ng/µL and stored at -80°C until required. qPCR analysis was performed on an ABI 7000 Sequence Detector using SYBR Green (Applied Biosystems). Data were normalized to *Hprt1* and relative expression was calculated using the $\Delta\Delta C_t$ method[6]. For epicardial genes, fold-change from baseline to d2 is highly under-estimated in our analysis, as epicardial markers are barely expressed in the uninjured adult heart and cycle threshold (C_t) values from those times points were too high to accurately derive expression level; therefore, fold change is shown relative to d2 values. The actual extent of re-activation and marker re-expression is better demonstrated at the protein level by immunofluorescence, however, qPCR is included to demonstrate temporal changes between d2-d21. Primer sequences were as follows:

Real time qRT-PCR Primers

Hprt1	F: TCAGTCAACGGGGGACATAAA<br>R: GGGGCTGTACTGCTTAACCAG
Acta2	F: GTCCCAGACATCAGGGAGTAA<br>R: TCGGATACTTCAGCGTCAGGA

Sm22α	F: CAACAAGGGTCCATCCTACGG<br>R: ATCTGGGCGGCCTACATCA
Pecam-1	F: CTGCCAGTCCGAAAATGGAAC<br>R: CTTCATCCACCGGGGCTATC
Aldh1a2	F: TGAGTTTTGGCTTACGGGAGT<br>R: TTGTTGTGAGGCAAGAGTGG
Wt-1	F: TTCAAGGACTGCGAGAGAAG<br>R: GGGAAAACTTTCGCTGACAA
Tbx18	F: ACGAAATAGGCACCGAGATG<br>R: TTGTCCACCGGCACAATATC
Tcf21	F: CGACAAGTACGAGAACGGTTAC<br>R: TGTAGTTCCACACAAGCGGTT
Tmsb4x	F: ATGTCTGACAAACCCGATATGGC<br>R: CCAGCTTGCTTCTCTTGTTCA
Snai1	F: CTTGTGTCTGCACGACCTGT<br>R: CAGGAGAATGGCTTCTCACC
Snai2	F: CATTGCCTTGTGTCTGCAAG<br>R: CAGTGAGGGCAAGAGAAAGG
Emcn	F: CTCCCGAAGGAACGACCAAA<br>R: AGGGGACCTTCAGTTGTTGT
Cspg4 (NG2)	F: GGGCTGTGCTGTCTGTTGA<br>R: TGATTCCCTTCAGGTAAGGCA

Adult Epicardial Explants

Culture dishes were pre-coated with 0.1% gelatine and air dried. Post MI hearts (d2) were collected in explant medium (a 1:1 mixture of low glucose Dulbecco's modified Eagle's medium and Medium 199 with 15% ES FCS, 1 % Glutamax and 1% non-essential amino acids; all from Invitrogen) and placed in PBS with 1% penicillin/streptomycin while the epicardium of each heart was carefully peeled away, taking care to separate AVS and myocardial portions into separate wells of a 6 well plate. Coverslips were placed on top of the tissues and pressed down to encourage adhesion. Explants were air dried at 37°C for 30 minutes and then explant medium was added to cover the sample, which was incubated at 37°C and monitored daily.

Statistics

Power calculations, based on our previous studies (e.g.[7]), determined that a minimum of 5 animals per time point were required to assess the extent of epicardial activation and *de novo* vessel formation. Given the known mortality of ~10-15% associated with the LAD ligation model and variability in the extent of injury achieved, we aimed to collect 8 hearts per time point from 10 infarcted animals. As described above, pre-defined exclusion criteria were used to exclude samples in which infarcts were outside the range of 20-50% of LV myocardium. As this was primarily an observational study, with readouts that could, on the whole,

not have been anticipated in advance, power calculations could not be strictly applied to every aspect. After the initial observational analyses were performed on hearts from a time course series post-MI, we focussed heavily on the d7 time point as that in which coronary sinus sprouting, epicardial activation and formation of subendocardial vessels were maximally observed. We retrospectively increased sample size for the d7 time point to ensure robustness and reproducibility of the largely descriptive readouts. Blinding was only used for assessment of coronary sinus sprouting, where images were reassessed and scored for sprouting by an independent observer whilst blinded to treatment (MI vs sham). Randomisation of animals to sham vs MI groups was determined by the surgeon at the time of LAD ligation.

Statistical analyses were performed with GraphPad Prism Software. For the quantitative comparison of Pdgfb-EYFP+ECs pre/post-MI, a two-tailed unpaired Student's t-test was used to determine any significant differences. The requirements for a t-test were assessed using a Shapiro-Wilk test for normality and an F-Test to compare the variances. For the comparison of subendocardial vessel density, a one-way ANOVA with Bonferroni correction was used. Significance is indicated in the figures, as follows: *: $p \leq 0.05$; **: $p \leq 0.01$; ***: $p \leq 0.001$.

REFERENCES

1. Claxton S, et al. Efficient, inducible Cre-recombinase activation in vascular endothelium. *Genesis*. 2008;46(2):74-80.

2. Srinivas S, et al. Cre reporter strains produced by targeted insertion of EYFP and ECFP into the ROSA26 locus. *BMCDevBiol*. 2001;1(4).

3. Miquerol L, et al. Architectural and functional asymmetry of the His-Purkinje system of the murine heart. *Cardiovascular Research*. 2004;63(1):77-86.

4. Zhou B, et al. Epicardial progenitors contribute to the cardiomyocyte lineage in the developing heart. *Nature*. 2008;454(7200):109-13.

5. Rossdeutsch A, Smart N, Dube KN, Turner M, and Riley PR. Essential role for thymosin beta4 in regulating vascular smooth muscle cell development and vessel wall stability. *Circ Res*. 2012;111(4):e89-102.

6. Livak KJ, and Schmittgen TD. Analysis of relative gene expression data using real-time quantitative PCR and the 2(-Delta Delta C(T)) Method. *Methods*. 2001;25(4):402-8.

7. Smart N, et al. De novo cardiomyocytes from within the activated adult heart after injury. *Nature*. 2011;474(640-4.

SUPPLEMENTAL FIGURES

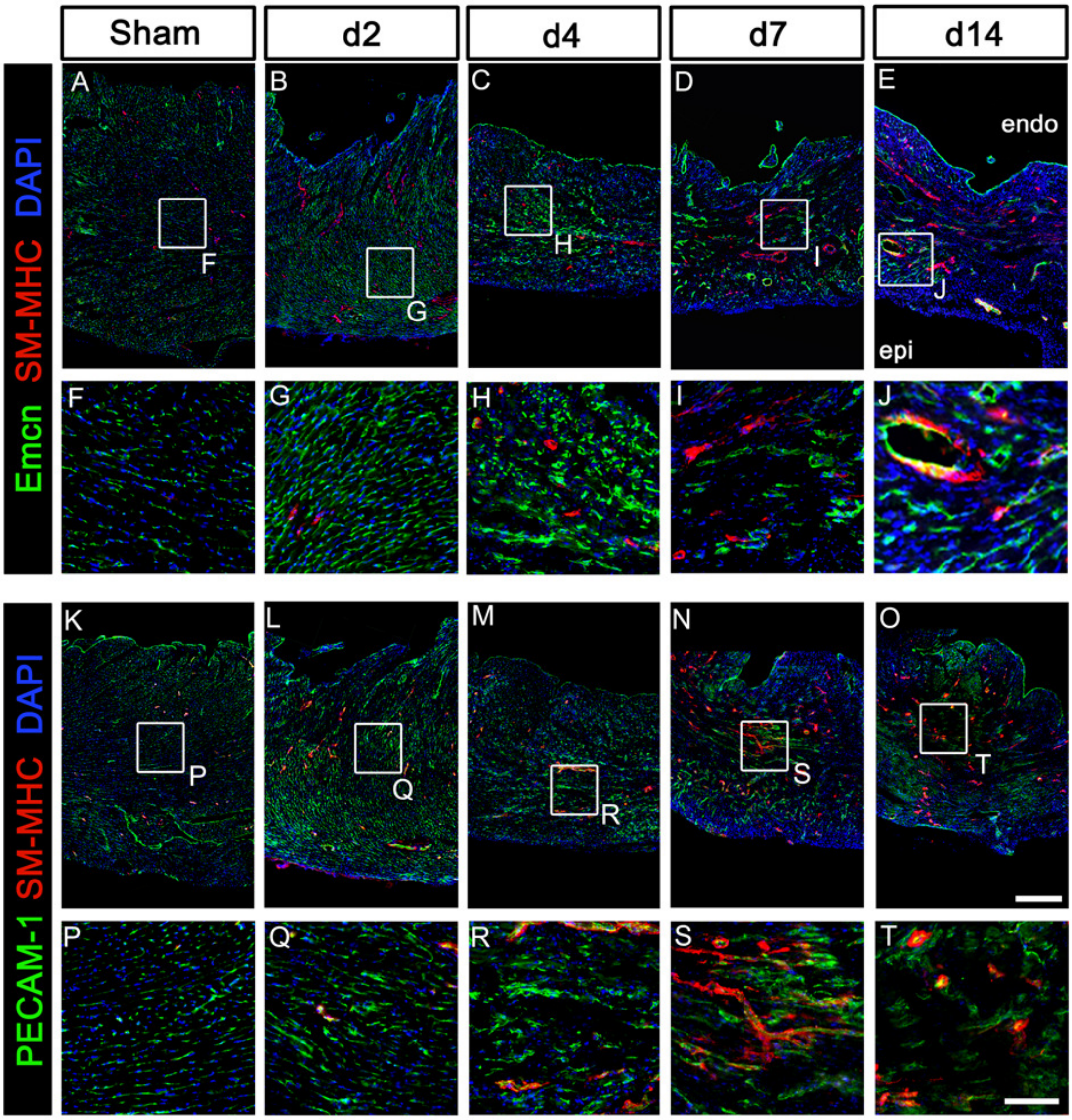

Supplemental Figure 1. Temporal remodelling of the capillary network into the infarcted myocardium. Visualised by Emcn/SM-MHC (**A**-**J**) or PECAM-1/SM-MHC (**K**-**T**) immunofluorescence; boxed regions in A-E; K-O enlarged in the respective panels below in F-J; P-T. Emcn (**B**, **G**) and PECAM-1 (**L**,**Q**) were up-regulated in capillary endothelial cells by d2 post-MI, compared with the respective sham (**A**, **F**, **K**, **P**). The organisation of the capillary network (as seen in sham) was disrupted, with branching apparent by d4 (**C**, **H**, **M**, **R**) and further increased by d7 (**D**, **I**, **N**, **S**). Sprouting and expansion into the infarct/border zone occurred despite a noticeable reduction overall in capillary number within the ischemic region. Vessel remodelling over this period involves branching, enlarging and acquisition of smooth muscle support (temporal transitions in **F**-**J** and **P**-**T**). Representative sections of n=8 hearts at each time point and 4 sham hearts, imaged at d7). endo – endocardium; epi-epicardium. Scale bars: 500µm (O, applies to A-E; K-O); 100µm (T, applies to F-J; P-T)

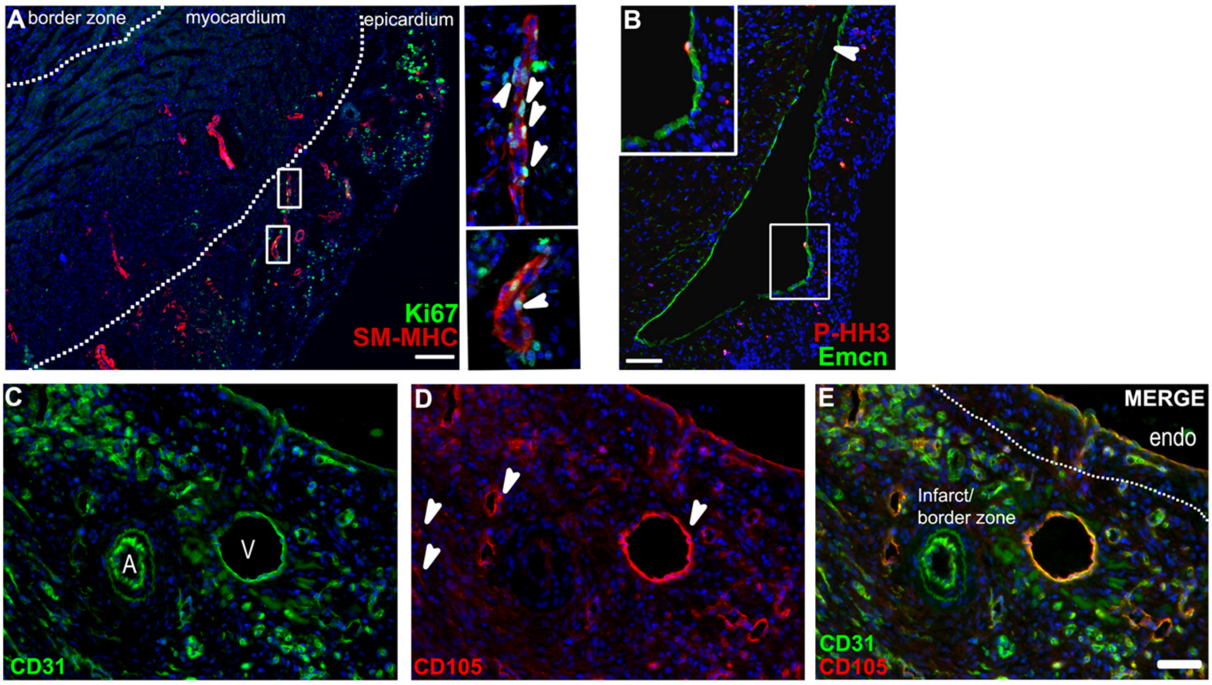

Supplemental Figure 2. Intrinsic neovascularisation includes a role for angiogenesis but most proliferating vascular cells are found in the activated epicardium.
In support of an angiogenic contribution, proliferating endothelial and smooth muscle cells are readily detected by immunofluorescence in the heart post-myocardial infarction but these are predominantly found within the newly formed epicardial network (**A**, enlarged insets correspond to boxed regions) or, rarely, in venous endothelial cells in regions of high epicardial activity (**B**). Endoglin expression was induced in veins, but not arteries, within the infarct region and surrounding border zone, supporting a greater tendency for angiogenesis, from veins (**C-E**), distinguishable morphologically after PECAM-1 immunofluorescence (**C**). A – artery; V – vein; endo – endocardium. Scale bars: 200µm (A); 100µm (B); 50µm (C-E).

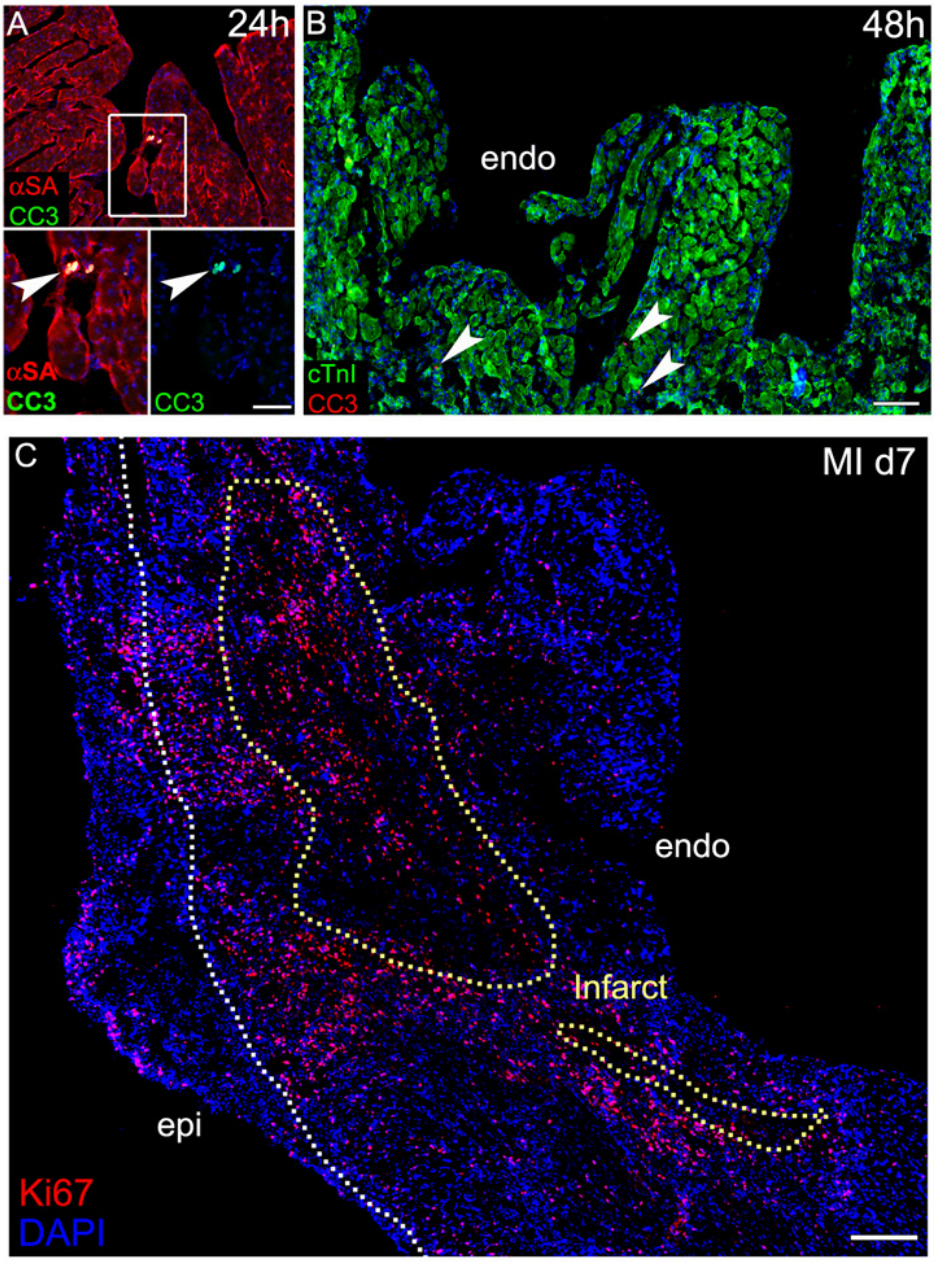

Supplemental Figure 3. Morphological changes appear to facilitate formation of trabeculae post-myocardial infarction, with only a modest degree of apoptosis and proliferation. Rare clusters of cleaved caspase 3 (CC3)+ cardiomyocytes were detected by immunofluorescence (arrowheads, **A-B**), adjacent to forming protrusions. While this suggested a possible role for apoptosis in creating endocardial lumina, the scarcity of CC3+ cells in trabeculating regions (0.56±0.07% at 24h (**A**); 0.23 ± 0.09% at 48h (**B**) and declining thereafter (not shown); n = 8 hearts per time point) suggests that this is not a predominant mechanism of trabecular formation. High levels of proliferation were observed in the infarct region and border zone, as well as in the activated epicardium, during the time of its expansion (**C**). Despite the extensive remodelling observed at the endocardial surface, proliferation rates were relatively low. Dotted white line indicates the epicardial-myocardial boundary; dotted yellow line outlines the infarcted regions. epi – epicardium; endo- endocardium. Scale bars: A: 20μm; B: 50μm; C:200μm.

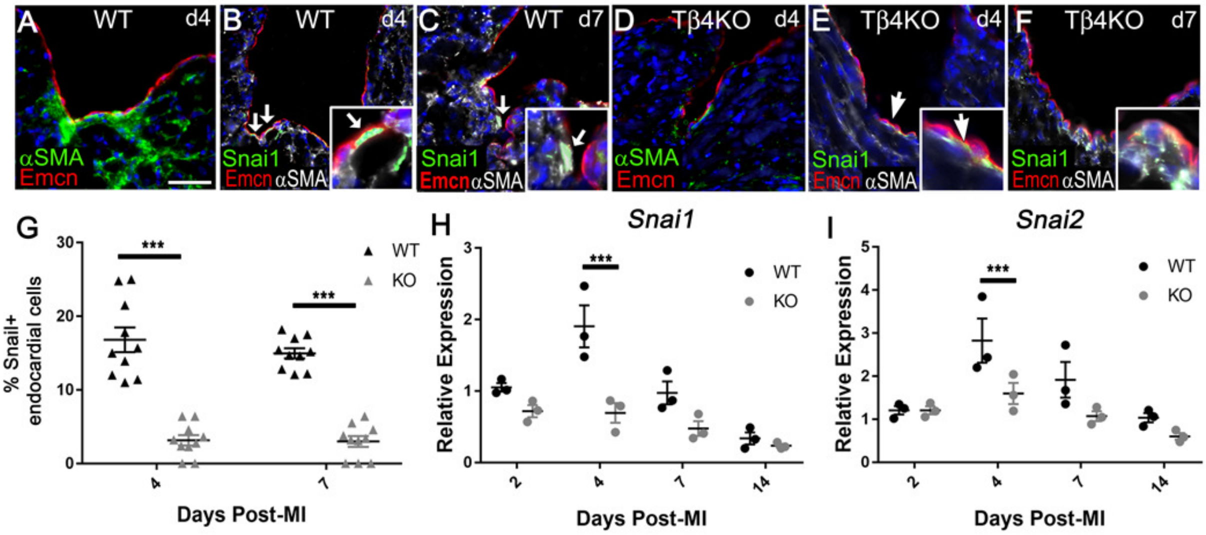

Supplemental Figure 4. Diminished expression of *Snai*1/2, regulators of endothelial-mesenchymal transition, in Thymosin β4 KO endocardium. The reduction in delaminating αSMA+ cells (Figure 11) was accompanied by reduced Snai1 expression, consistent with a possible endothelial-mesenchymal transition defect. Comparison of WT (**A**-**C**) and KO (**D**-**F**) revealed a significant reduction in the number of Snai1+ endocardial cells, quantified in **G** (each data point represents a separate heart with mean ± SEM, mean of 3 sections per heart, n=10 hearts per genotype; two-way ANOVA with Sidak's correction for multiple comparisons). qPCR confirmed reduced expression of *Snai1* (**H**) and *Snai2* (**I**); two-way ANOVA with Bonferroni correction for multiple comparisons; ***: $p<0.001$. Scale bar: 20µm (A, applies A-F).

SUPPLEMENTAL TABLE 1

Capillaries	Arteries	Veins	Endocardium	Subendocardial Vessels
Endomucin	Endomucin	Endomucin	Endomucin	Endomucin
Endoglin	Endoglin	Endoglin	Endoglin	Endoglin
Cx40-EGFP	Cx40-EGFP	Cx40-EGFP	Cx40-EGFP	Cx40-EGFP
VEGFR2	VEGFR2	VEGFR2	VEGFR2	VEGFR2
*Pdgfb*CreERT2	*Pdgfb*CreERT2	*Pdgfb*CreERT2	*Pdgfb*CreERT2	*Pdgfb*CreERT2

Table 1. The distinct marker profile of sub-endocardial vessels post-MI was shared with the endocardium, but not with ECs of capillaries, veins or arteries, consistent with, and suggestive of, their derivation from, the endocardium (green – expressed; yellow – weakly expressed; red – not expressed).
